# *Azadirachta indica* A. Juss Ameliorates Mouse Hepatitis Virus-Induced Neuroinflammatory Demyelination by Modulating Cell-to-Cell Fusion in an Experimental Animal Model of Multiple Sclerosis

**DOI:** 10.3389/fncel.2020.00116

**Published:** 2020-05-12

**Authors:** Lucky Sarkar, Ravi Kiran Putchala, Abass Alao Safiriyu, Jayasri Das Sarma

**Affiliations:** Department of Biological Sciences, Indian Institute of Science Education and Research Kolkata, Mohanpur, India

**Keywords:** *Azadirachta indica*, mouse hepatitis virus, cell-to-cell fusion and syncytia, meningoencephalomyelitis, neuroinflammatory demyelination, neuroprotective

## Abstract

Mouse hepatitis virus (MHV)-induced murine neuroinflammation serves as a model to study acute meningoencephalomyelitis, hepatitis, and chronic neuroinflammatory demyelination; which mimics certain pathologies of the human neurologic disease, multiple sclerosis (MS). MHV-induced acute neuroinflammation occurs due to direct glial cell dystrophy instigated by central nervous system (CNS)-resident microglia and astrocytes, in contrast to peripheral CD4+T cell-mediated myelin damage prevalent in the experimental autoimmune encephalomyelitis (EAE) model of MS. Viral envelope Spike glycoprotein-mediated cell-to-cell fusion is an essential mechanistic step for MHV-induced CNS pathogenicity. Although *Azadirachta indica* (Neem), a traditional phytomedicine, is known for its anti-inflammatory, anti-fungal, and spermicidal activities, not much is known about anti-neuroinflammatory properties of its bark (NBE) in MHV-induced acute neuroinflammation and chronic demyelination. Recombinant demyelinating MHV strain (RSA59) was preincubated with NBE to arrest the infection-initiation event, and its effect on viral replication, viral transcription, cytokine expression, and successive pathogenicity were investigated *in vitro* and *in vivo*. Virus-free Luciferase assay explained NBE’s anti-virus-to-cell fusion activity *in vitro*. Intracranial inoculation of RSA59 preincubated with NBE into the mouse brain significantly reduces acute hepatitis, meningoencephalomyelitis, and chronic progressive demyelination. Additionally, NBE effectively restricts viral entry, dissemination in CNS, viral replication, viral transcription, and expression of the viral nucleocapsid and inflammatory cytokines. From mechanistic standpoints, RSA59 preincubated with NBE reduced viral entry, viral replication and cell-to-cell fusion, as a mode of viral dissemination. Moreover, intraperitoneal injection with NBE (25 mg/kg B.W.) into mice revealed a significant reduction in viral Nucleocapsid protein expression *in vivo*. Conclusively, *A. indica* bark extract may directly bind to the virus-host attachment Spike glycoprotein and suppresses MHV-induced neuroinflammation and neuropathogenesis by inhibiting cell-to-cell fusion and viral replication. Further studies will focus on combining bioanalytical assays to isolate potential NBE bioactive compound(s) that contribute towards the anti-viral activity of NBE.

## Introduction

Neuroinflammatory cascades play an important defensive role against various pathogenic stimuli, toxins, ischemic injury, and protein accumulation that induce neurodegeneration and can challenge the host immune system. Inflammatory mediators [chemokines, Reactive oxygen species (ROS), proinflammatory and anti-inflammatory cytokines like Interleukin 6 (IL-6), Interleukin 10 (IL-10)] are produced by central nervous system (CNS) resident cells (microglia, astrocytes) or cells migrating from the peripheral blood and cause impairment of blood-brain barrier (BBB) integrity. Activated-microglia mediated neuroinflammation is a common pathological hallmark of various neurodegenerative diseases like Alzheimer’s disease (AD), Parkinson’s disease (PD), multiple sclerosis (MS), and cerebral ischemia (Milatovic et al., 2011; Tohidpour et al., 2017). Clinical studies show that infectious agents can cause inflammation of the CNS. Mouse hepatitis virus (MHV) belongs to the β-coronavirus group and is a prototype m-CoV. MHV (m-CoV) mainly infects mouse and induces acute hepatitis, meningitis, encephalitis, myelitis, and chronic phase progressive demyelination concurrent with axonal loss and serves as an experimental model for human neurological disease multiple sclerosis (MS; Bjartmar et al., 2003). I.C. inoculation with neurotropic MHV strains induces a biphasic neurological disease characterized by acute hepatitis and meningoencephalitis that precede the onset of chronic demyelination (Lavi et al., 1984b; Houtman and Fleming, 1996). Viral-induced inflammation (meningitis, encephalitis, and myelitis) reaches its peak at 6 days p.i. Viral titer reduces after day 7 p.i. but viral-induced inflammation initiates more complex adaptive immune responses leading to neuronal degeneration and axonal transection, inducing long-term neurological disorders at 30 days p.i. MS is commonly studied in experimental autoimmune encephalitis (EAE) models which demonstrate that myelin-specific CD4+T cells cause neuroinflammation and subsequent demyelination. Though this model is well-established, it is unable to dissect direct viral infection-induced myelin damage as opposed to CD4+T cell-mediated pathology. In this context, MHV-induced neuroinflammatory demyelination is considered a unique experimental animal model to study the role of direct neural cell death and damage, which may contribute to axonal loss and myelin damage as an inside-out model of demyelination (Weiner, 1973; Lavi et al., 1984a).

Traditional herbs find paramount importance in modern drug discovery. Regardless of significant advances in current medicine, the development of innovative therapeutic approaches is increasing rapidly. Conventionally, several non-steroidal anti-inflammatory drugs (NSAIDs) are used in treating neurodegenerative diseases. Most chronic diseases are multi-genic, and these mono-targeted drugs are unlikely to be effective due to growing drug resistance and the presence of side effects (Okpanyi and Ezeukwu, 1981; Mauriz et al., 2014; Thell et al., 2016) when consumed for long periods. In contrast, almost 80% of all herbal drugs (viz. nutraceuticals) designed by “Mother Nature” are highly effective and multi-targeted, but also often exert few side effects due to the composition and extraction procedure (Pérez-Hernández et al., 2016; Rasool et al., 2014).

*Azadirachta indica* A. Juss (Neem), an ethnomedicinal plant belonging to class: Dicotyledonous; order: Fagales; family: Meliaceae; is indigenous to African and Asian folk medicine (Pankaj et al., 2011; Jhariya et al., 2013; Alzohairy, 2016). Neem bark extract (NBE) was reported to possess anti-inflammatory, anti-allergenic, anti-immunomodulatory, anti-tumor (Gallic acid, (-) Epicatechin, Catechin, Margolone, Isomergolonone), anti-fungal, anti-dermal (Nimbidin), anti-protozoal and spermicidal properties (Manogaran et al., 1998; Biswas et al., 2002; Akihisa et al., 2009; Ghimeray et al., 2009; Pandey et al., 2014; Vinoth et al., 2012). NBE showed potential antibacterial activity against *Salmonella paratyphi* and *S. Typhi* (Panchal et al., 2013; Al Akeel et al., 2015), and hepatoprotective activity against CCl_4_-induced hepatic damage in albino rats (Gomase et al., 2011; Bucur et al., 2014) with strong evidence of anti-oxidant properties. Interestingly, NBE is also reported to block the entry of HSV1 (Herpes Simplex virus; Tiwari et al., 2006, 2010). While NBE is shown to diminish the effects of malaria on cerebellar Purkinje cells in *Plasmodium berghei*-infected mice, very little is known about its anti-neuroinflammatory mechanism (Lucantoni et al., 2010; Bedri et al., 2013).

MS is a multifactorial neuroinflammatory disease. The MHV-induced model of MS is unique because the direct viral infection causes microglial activation and sets the stage for chronic demyelination. The current study is focused on understanding the potential effect of NBE in MHV-induced neuroinflammatory demyelination and demonstrates for the first time that direct binding of NBE with the intact virus can ameliorate neuroinflammation. NBE is known for its numerous anti-microbial, anti-inflammatory properties but rarely known for its anti-neuroinflammatory role. Several studies have used phytochemicals to target inflammation *per se*, but so far, very few reports show a significant reduction in direct binding of the virus to the cell causing a reduction in cell-to-cell fusion. One of the vital proteins responsible for virus-cell fusogenicity is the Spike envelope glycoprotein. Recently, our group showed that two characteristic internal prolines in the hydrophobic fusion peptide stabilizes Spike protein and play an imperative role in viral entry, viral dissemination, and syncytia formation *in vitro*. *In vivo*, transcranial inoculation with proline mutant viruses delineated altered neuropathogenicity as a result of impaired viral infectivity and consecutive fusogenicity (Sadasivan et al., 2017; Singh et al., 2019). Henceforth, it is tempting to speculate that spike protein-mediated cell-to-cell fusion may be a potential checkpoint to reduce neuropathogenesis using specific nutraceuticals (i.e., NBE). We, therefore, investigated anti-virus-cell fusion properties of NBE, and report for the first time that preincubation of the virus with NBE significantly lowers viral infectivity and the resultant neuropathology *in vitro* and *in vivo*.

## Materials and Methods

### Laboratory Preparation of *A. indica* (Neem) Bark Extract; NBE

Air-dried bark of the neem tree was ground well in a mortar, and 1 kg bark powder was dissolved in 1.5 L methanol by maceration for 1 week. The suspension was mixed vigorously in a shaker at 25°C for 24 h. The extract was collected by filtering through Grade 1 Whatmann™ filter paper and dried using a rotary vacuum evaporator at 55°C (Alam et al., 2010; Nelson et al., 2016). This lyophilized fine brown powder (crude bark extract) was dissolved in Dimethyl sulfoxide (DMSO; cell-culture grade) at a concentration of 100 mg/ml followed by filtration through a 0.22 μm membrane filter and stored in the freezer at −20°C (Schumacher et al., 2011; Nelson et al., 2016). NBE raw powder was a kind gift from Dr. Mahadeb Pal (Bose Institute, Kolkata). The working concentrations (50–1,000 μg/ml) were prepared by dilution in cell culture media (*in vitro*) or 1 × PBS (*in vivo*).

### Cell Lines

The *in vitro* study used two murine cell lines, L2 rat fibroblast cell line (American Type Culture Collection, ATCC, RRID:CVCL_0383) and Neuro-2A neuroblastoma cell line (Kind gift from Dr. Anirban Basu NBRC, Haryana India, ATCC, RRID:CVCL_0470). L2 cells were cultured and maintained in 1 × L2 medium (Dulbecco’s Modified Eagle Medium) supplemented with 10% Fetal Bovine Serum (FBS) and 1% Penicillin (10,000 μ/ml)-Streptomycin (100 mg/ml) antibiotic cocktail, 1% 10 mM HEPES buffer solution (Invitrogen, Thermo Fisher Scientific, Waltham, MA, USA), 7.5% NaHCO_3_ and 0.1% L-glutamine. Neuro-2A cells were maintained in Minimum Essential Medium (MEM) supplemented with 10% FBS and 1% Penicillin-Streptomycin antibiotic cocktail. All cell culture media and reagents were supplied from Gibco, Thermo Fisher Scientific, Waltham, MA, USA. For the cell-to-cell fusion assay, HeLa; human cervical cancer cell line (ATCC, RRID:CVCL_0030) and BHK-R; Baby Hamster Kidney cells (obtained from Dr. Susan Weiss laboratory, University of Pennsylvania, Philadelphia, PA, USA) were stably transfected with MHVR_1_, functional receptor for murine coronavirus MHV-A59. Both HeLa and BHK-R cells were maintained in DMEM media supplemented with 10% FBS and 1% Penicillin-Streptomycin antibiotic cocktail. 100 μg/ml G418 antibiotic was added with 10% FBS containing DMEM to BHK-R cells. All cells were grown as an adherent monolayer till confluence, and respective experiments were performed.

### Viruses

A neurotropic demyelinating strain of MHV, MHV-A59 (Lavi et al., 1984b; Das Sarma et al., 2000), and its isogenic recombinant strain, RSA59, were used to infect mice and cell lines. The MHV spike gene was introduced by replacing non-essential genes 4A and part of 4B by targeted RNA recombination in the RSA59 strain (Das Sarma et al., 2000, 2002, 2008). RSA59 also expresses enhanced green fluorescence protein (EGFP) which is useful to trace viral entry and dissemination through cells and tissues.

### Plasmids

Plasmid pT7EMCLuc (Gift from Vaibhav Tiwari, Midwestern University, Downers Grove, IL, United States) expresses the firefly luciferase gene under the T7 promoter, pMH54_EGFP_ is a Spike-expressing plasmid (PP, two proline residues in cell-to-cell fusion domain; Das Sarma et al., 2002, 2008; Singh et al., 2019), and pCAGT7 expresses T7 RNA polymerase with the chicken actin promoter and the CMV enhancer.

### Chemicals and Reagents

MTT reagent Thiazolyl Blue Tetrazolium Bromide (Sigma–Aldrich), All cell culture dishes (Nunc), 4′,6-diamidino-2-phenylindole (DAPI; VectaShield, Vector Laboratories); Trizol (Ambion), DEPC, Diethyl pyrocarbonate (Ambion), RNAlater stabilizing solution (Ambion-Thermo Fisher Scientific), High-Capacity Reverse Transcription Kit (Applied Biosciences, Inc. Foster, CA, USA), DyNAmo ColorFlash SYBR Green qPCR kit (Thermo Fisher Scientific), EDTA-free Protease-cocktail inhibitor (Roche Mannheim Germany), Pierce^®^ BCA protein assay kit (Thermo Fisher Scientific, Rockford, IL, USA), polyvinylidene difluoride membranes (Millipore, Bedford, MA, USA), Methanol (SRL), γ-actin polyclonal antibody (BioBharati LifeScience Private Limited, BB-AB0025), Horseradish peroxidase (HRP)-conjugated secondary IgG antibody (Goat anti-Rabbit, Jackson Immuno Research, Cat no. 111-035-003, RRID:AB_2313567), Horseradish peroxidase (HRP)-conjugated secondary IgG antibody (Goat anti-Mouse, Jackson Immuno Research, Cat no. 115-035-146, RRID:AB_2307392), SuperSignal WestPico chemiluminescent substrate (Thermo Fisher Scientific, Rockford, IL, USA), Agarose (Invitrogen by Life Technologies), Neutral red (Sigma–Adrich, St. Louis, MO, USA), Ketamine (Troikaa Pharmaceuticals Limited, Xylazine Indian Immunologicals Limited), Gelatin (Merck), PFA, Paraformaldehyde (Merck), Anti-Iba1 (Wako, Richmond, VA, USA, Cat no. 019-19741, RRID:AB_839504) antibody, Avidin-biotin immunoperoxidase technique (Vector Laboratories), Refrax mounting medium (Anatech Limited, MI, USA).

### *In vitro* Cell Viability Assay

For cell viability characterization, MTT assay was performed in the Neuro-2A cell line. Cells were seeded in 96-well plates at 1 × 10^4^ cells/well and maintained for 24 h at 37°C in 10% FBS containing MEM medium. Cells were treated with NBE in a concentration range of 50–1,000 μg/ml (primary stock- 100 mg/ml) and examined at 12 h and 24 h p.i. The media was discarded, cells were washed with 1 × PBS and 200 μl of fresh media containing (5 mg/ml) of MTT (Thiazolyl Blue Tetrazolium Bromide) was added to each well and incubated at 37°C with 5% CO_2_ for 5 h. The entire solution was discarded carefully without disturbing the purple-colored formazan crystals formed on the surface of the well. Hundred micro-liter of DMSO was added to each well, and absorbance was measured at 570 nm using an Absorbance Plate Reader (Hussain et al., 2018). Net survival values were calculated and plotted using GraphPad Prism 6.0., Maximum non-cytotoxic concentration (MNCC) dose and effective concentration (EC_50_) of NBE were determined in Neuro-2A cells. All experimental analyses were performed all-time in a day. The maximum number of passages for cell lines used in this study is 4.

### NBE Treatment in RSA59 Infected Neuro-2A Cells

Neuro-2A cells were cultured, grown as a monolayer till confluence (70–80%), and infected with RSA59 (MOI 1) in 2% DMEM infection medium and incubated with virus in intermittent shaking for a 15 min interval following which the virus inoculum was discarded, and fresh 10% MEM media containing 200 μg/ml NBE was added to the cells and incubated at 37°C in 5% CO_2_ till 12 h p.i. for experimental analysis. In parallel, RSA59-infected controls were maintained in separate Neuro-2A cell cultures.

### Virus-Free Cell-to-Cell Fusion Assay

The cell-to-cell fusion assay was executed with slight modifications of previously established methodology (Tiwari et al., 2010; Ou et al., 2016). Wild-type HeLa cells, considered as “effector” cells for this experiment were co-transfected with plasmid pT7EMCLuc expressing the firefly luciferase gene under the T7 promoter as well as the spike-expressing plasmid pMH54_EGFP._ BHK-R cells in continuous culture, used as “target” cells were transfected with pCAGT7 expressing T7 RNA polymerase with the chicken actin promoter and the CMV enhancer. Effector cells were then preincubated with 200 μg/ml NBE for 25 min at 36 h post-transfection. In a parallel experiment effector cells were also treated with vehicle (DMSO).

For fusion at 36 h post-transfection, effector and target cells were trypsinized and co-cultured in a 1:1 ratio in 24-well culture plates. The untreated pT7EMCLuc-expressing effector cells and only T7 RNA polymerase-transfected target cells were used as negative controls (vector control) and untreated effector cells expressing both pT7EMCLuc and pMH54_EGFP_ plasmids were used as positive controls. The luciferase reporter assay (Promega, Madison, WI, USA) was performed to estimate the luciferase activity at 36 h post-co culture as described previously. Luciferase activity is considered as a measurement of cell-to-cell fusion ability and is directly correlated with the fusion property of the spike gene. Luciferase reporter activity was plotted using GraphPad Prism 6.0 software. Level of significance between untreated positive control (pT7EMCLuc + pMH54_EGFP_ plasmids + no NBE), NBE treated co-cultured sets (pT7EMCLuc + pMH54_EGFP_ plasmids + 200 μg/ml NBE), and vehicle (DMSO) treated cells was calculated and compared using unpaired student’s *t*-test and RM One-way ANOVA test followed by Tukey’s multiple comparison test.

### Infection of Neuro-2A Cells With RSA59 Preincubated With NBE

Neuro-2A cells were cultured and grown as a monolayer till confluence. RSA59 virus (MOI = 1) was preincubated with NBE (200 μg/ml) for 15 min at 4°C. Then the virus was used to infect Neuro-2A cells in infection medium containing 2% FBS and cells were incubated with the virus for 1 h 15 min with intermittent shaking for a 15 min interval following which the virus inoculum was discarded, and fresh 10% MEM media was added to the cells and incubated at 37°C with 5% CO_2_ for 10, 12, and 15 h for subsequent analyses. The experimental sets are C, Control (2% DMEM media); OV, Only-virus infected [RSA59 (MOI 1) in 2% DMEM]; V, Infected-vehicle control [RSA59 (MOI 1) in 2% DMEM + DMSO], and T, NBE treated (200 μg/ml) [RSA59 (MOI 1) in 2% DMEM media + NBE in DMSO].

### Viral Plaque Assay to Determine the Viral Titer *in vitro*

The viral titer of the supernatants collected from OV (only-virus infected), V (infected-vehicle) and T (NBE treated) cell cultures at 10, 12, and 15 h p.i. were performed by standard plaque assay. Briefly, L2 cells were seeded in 6-well plates. The supernatant was serially diluted in 2% FBS containing media. L2 monolayers were infected with 200 μl of diluted virus followed by 1 h incubation at 37°C, and 5% CO_2_ with intermittent rocking every 15 min. Then, cells were overlaid with 1 volume of 1.4% dissolved agarose in 1 × PBS with 1 volume of 2 × L2 media allowed to solidify. After 18–20 h, each well was overlaid by 1.4% agarose having 0.04% neutral red (60 μl per well), and plaques were manually counted after 5 h of incubation (Das Sarma et al., 2008; Kishore et al., 2013). Viral titer was calculated as plaque-forming units (PFU) based on a mathematical formula = (Number of plaques *dilution factor/ml), and the logarithmic values of the titers were plotted in GraphPad Prism 6.0 as a scatter diagram.

### RNA Isolation, cDNA Preparation, and Real-Time Quantitative Polymerase Chain Reaction (q-PCR) Analysis *in vitro*

RNA isolation was performed from OV, V, and T cultures using a standard Trizol protocol (Ambion). The concentration of RNA was measured using a Thermo Fisher Scientific NanoDrop 2000/2000c Spectrophotometer. One microgram of RNA was used for cDNA preparation using the High-Capacity Reverse Transcription Kit protocol (Applied Biosciences, Inc. Foster, CA, USA).

Quantitative Real-time PCR analysis was performed using DyNAmo ColorFlash SYBR Green qPCR kit (Thermo Fisher Scientific) in a Step One plus Real-time PCR system (Thermo Fisher Scientific) under the following conditions: initial denaturation at 95°C for 7 min, 40 cycles of 95°C for 10 s, 60°C for 30 s, melting curve analysis at 60°C for 30 s. The following primer pairs were designed and used: GAPDH gene forward-5′GCCCCTTCTGCCGATGC3′, reverse-5′CTTTCCAGAGGGGCCATCC3′; viral Nucleocapsid gene (IZJ) forward-5′AGGATAGAAGTCTGTTGGCTCA3′, reverse-5′GAGAGAAGTTAGCAAGGTCCTACG3′; Spike (viral S gene) forward-5′ GCCAGTATACCATTTGTCTGTTACCT3′, reverse-5′CTACTACGTTTTTGTTTAG3′; proinflammatory cytokines, IL-6 gene forward-5′AGTTGCCTTCTTGGGACTGA3′, reverse-5′TCCACGATTTCCCAGAGAAC3′; and IL-10 gene forward-5′ AGTGGAGCAGGTGAAGAGTG3′, reverse-5′TTCGGGAGAGGTACAAACG3′. Reactions were performed in quintuple (*n* = 5). C_t_ values were calculated and were normalized with GAPDH C_t_ values. Relative quantitation was achieved using the comparative threshold (ΔCΔ_t_) method and plotted. The levels of mRNA expression of target genes were normalized with the GAPDH gene and expressed as relative fold change (2^−ΔΔCt^) compared to their respective infected-vehicle controls (V).

### Protein Isolation From Cells and Western Blot Analysis *in vitro*

Total protein was obtained from C, OV, V, and T cultures using a standard protein extraction protocol. Briefly, cells were scraped from the culture dish, washed in ice-cold PBS and centrifuged at 1,500 r.p.m. for 7 min at 4°C in an Eppendorf 5415R. The obtained cell pellet was resuspended in ice-cold protein extraction RIPA buffer [50mM Tris Base (pH 7.6), 150 mM NaCl, 1% Triton X-100, 0.1% SDS, 0.5% Sodium deoxycholate] containing EDTA-free Protease-cocktail inhibitor, and homogenized by mild vortexing at regular 15 min time intervals for 90 min. The lysed cell suspension was centrifuged at 4°C for 10 min at 13,600 r.p.m. The supernatant (total cell lysate) was separated in a fresh microcentrifuge tube, and concentration was estimated using the Pierce^®^ BCA protein assay kit (Thermo Fisher Scientific, Rockford, IL, USA).

Fifteen microgram of each protein extract was resolved on a 12% polyacrylamide gel followed by transfer onto polyvinylidene difluoride membranes (Millipore, Bedford, MA, USA) in transfer buffer (25 mM Tris, 192 mM glycine and 20% methanol). The membrane was subsequently blocked with 5% non-fat skimmed milk in TBST (Tris-buffered saline containing 0.1% v/v Tween-20) for 1 h at room temperature followed by incubation in primary monoclonal antibody directed against viral nucleocapsid protein (N) of MHV-JHM at a dilution of 1:50 and γ-actin polyclonal antibody as an internal control (BioBharati LifeScience Private Limited, BB-AB0025) overnight at 4°C. The membrane containing protein samples was then washed with TBST and incubated with Horseradish peroxidase (HRP)-conjugated secondary IgG antibody; Goat anti-Mouse (Jackson Immuno Research) against viral nucleocapsid protein primary antibody and Goat anti-Rabbit (Jackson Immuno Research) against γ-actin primary antibody in blocking solution for 1 h at room temperature. The blots were subsequently washed in TBST solution, and the immunoreactive bands were visualized using SuperSignal WestPico chemiluminescent substrate (Thermo Fisher Scientific, Rockford, IL, USA). Non-saturated bands were visualized using Syngene G: box ™Chemidoc system using GENSys Software and densitometric analyses and quantification of immunoreactive bands were carried out using ImageJ software. Protein expression levels of target genes were normalized to γ-actin gene expression and expressed as relative fold change compared to their infected-vehicle controls.

### Animal Ethics Statement

Four-week-old, C57BL/6 male mice (obtained from Jackson Laboratory) were maintained at controlled environmental conditions (22 ± 2°C, 60 ± 5% humidity) with 12 h light/dark cycle in individually ventilated cages at IISER Kolkata small animal facility. They were provided with standard diet and water ad libitum. The studies involving animals were reviewed and approved by institutional animal care and use committee at the Indian Institute of Science Education and Research Kolkata (IISER-K). The animal protocols were adhered to the guidelines of the Committee for the purpose of control and supervision of experiments on animals (CPCSEA), India.

### Inoculation of Mice

Mice were anesthetized by intraperitoneal injection of 120–140 μl (depending on body weight) of 40% (v/v in distilled water) Ketamine (50 mg/ml) and 10% (v/v in distilled water) Xylazine mixture (20 mg/ml) in the ratio of Ketamine: Xylazine: distilled water = 4:1:4. Mice do not feel pain and claustrophobia while using Ketamine and Xylazine. Intracranial inoculation with parental demyelinating strain MHV-A59 and its recombinant strain RSA59 were performed in mice at 50% LD_50_ dose, i.e., 2,000 PFU and 20,000 PFU, respectively (Das Sarma et al., 2002, 2008). Twenty microliter of the diluted virus (in PBS + 0.75% BSA, bovine serum albumin) was injected into the right half of the cerebral hemisphere of each mouse. Mock-infected controls were inoculated similarly with PBS + 0.75% BSA and maintained in parallel. All mice were monitored daily for signs of morbidity and mortality. With an overdose of Ketamine and Xylazine, mice were deeply anesthetized and sacrificed to harvest the brain, liver, and spinal cords for histopathological and immunohistochemical analyses at acute (day 6 p.i.) and chronic stages (day 30 p.i.) of RSA59 infection. The experimental procedure was performed between 8 a.m. and 5 p.m.

### NBE Administration in C57BL/6 Mice

Primarily, mice were divided into four major groups- mock control; MI (PBS+BSA); only virus-infected, OV (virus in PBS-BSA); infected-vehicle control, V (virus in PBS-BSA + DMSO); and NBE treated, T (virus preincubated with NBE+ PBS-BSA). No randomization was performed, the mice were arbitrarily assigned for NBE treatment. RSA59 virus particles (20,000 PFU) were preincubated with NBE at a range of 10–50 mg/kg B.W.) or with DMSO (vehicle) for 40 min on ice (4°C). Twenty microliter of preincubated mixture was intracranially (i.c.) injected into respective groups of mice. 50 mg/kg B.W. of NBE appeared to be a lethal dose for mice and all mice died by day 1 p.i. Mice were physically active and healthy at 10, 25 and 35 mg/kg B.W. doses with no NBE toxicity. All animal experiments were conducted with the most effective non-lethal dose, i.e., 25 mg/kg B.W. (NLD). Daily, mouse body weights were measured till day 6 p.i. and observed for any physiological changes in behavior and activity. Mice were sacrificed at day 6 and day 30 p.i. for biochemical and pathological analyses. The total number of mice used was 125, and the initial number of mice used per group was three.

To test the efficacy of NBE when administered intraperitoneally, mice were given an i.p. injection of 25 mg/kg B.W. NBE in 100 μl 1 × PBS (Das et al., 2010; Somsak et al., 2015) before 24 h of intracranial infection with MHV-A59 (2,000 PFU, parental demyelinating MHV strain) and also in every 3 days interval until day 7 p.i. Mice were observed daily for clinical signs and symptoms and sacrificed at day 5 and 7 p.i. for experimentation.

### Estimation of Viral Replication From Tissue Samples

The efficiency of NBE (25 mg/kg B.W.) in controlling RSA59 viral replication in mice was determined by routine plaque assay. On day 6 p.i., OV, V, and T mice were euthanized and perfused transcardially with 20 ml of PBS. Brain, liver and spinal cord tissues were harvested aseptically for determination of viral titers and placed into 2, 3, and 1 ml of isotonic saline containing 0.167% gelatin (gel saline) respectively. All organs collected for viral titer were weighed and kept frozen at −80°C until titered. Tissues were subsequently homogenized using a Qiagen Tissue Ruptor, centrifuged at 3,000 r.p.m. for 7.5 min at 4°C and supernatants were collected and stored at −80°C until further use. Viral titer values were quantified by standard plaque assay protocol on tight monolayers of L2 cells infected with 250 μl of virus supernatant, and the rest of the procedure was followed as described previously (Das Sarma et al., 2008; Kishore et al., 2013).

### RNA Isolation and Real-Time Quantitative Polymerase Chain Reaction (q-PCR) Analysis *in vivo*

*In vivo*, following transcardial perfusion with DEPC treated 1 × PBS, brain tissues were collected in RNA later stabilizing solution and kept at −80°C. RNA was extracted from brain tissues of OV, V, and T mice at day 6 p.i. using Trizol reagent. The concentration of RNA was measured using a Thermo Fisher Scientific NanoDrop 2000/2000c Spectrophotometer. One micro-gram of RNA was used from each set for cDNA preparation using a High-Capacity Reverse Transcription Kit protocol (Applied Biosciences, Inc. Foster, CA, USA). Quantitative Real-time PCR analysis was performed using the DyNAmo ColorFlash SYBR Green qPCR kit (Thermo Fisher Scientific) in a Step One plus Real-time PCR system (Thermo Fisher Scientific); as described previously (*in vitro* section). The levels of mRNA expression of target genes were normalized with the GAPDH gene and expressed as relative fold change (2^−ΔΔCt^) compared to their respective infected-vehicle controls (V).

### Protein Extraction From Tissue Samples and Western Blot Analysis

Thirty milligram of brain and liver tissues were collected from mice following transcardial perfusion with 20 ml PBS and flash-frozen in liquid nitrogen. Each tissue was lysed in 1 ml of RIPA buffer containing EDTA-free Protease-cocktail inhibitor. Tissue samples were homogenized (Qiagen homogenizer) and sonicated thrice on the ice at 30% amplitude of 30 kHz for 30 s using a Sartorius Labsonic M sonicator. Tissue lysates were centrifuged at 13,600 r.p.m. for 30 min at 4°C. The supernatant of each sample was collected as whole protein extract, and concentration was estimated using a Pierce^®^ BCA protein assay kit. Proteins were resolved on 12% polyacrylamide gels followed by transferring onto polyvinylidene difluoride membranes and western blot analysis was performed as described previously (*in vitro* section).

### Histopathological and Immunohistochemical Analyses

Mice were sacrificed at days 6 and 30 p.i. and perfused transcardially with PBS followed by 1 × PBS containing 4% paraformaldehyde (PFA). Brain and liver tissues were collected, post-fixed in 4% PFA overnight, and embedded in paraffin (Das Sarma et al., 2008; Das Sarma, 2010; Chatterjee et al., 2013) Five micron thick sections of the paraffin-embedded tissues were prepared and stained with Hematoxylin & Eosin to evaluate acute inflammatory signs like encephalitis and meningitis along with hepatitis at day 6 p.i. At day 30 p.i. spinal cord tissue sections were stained with LFB to detect myelin damage.

All immunohistochemical analyses were performed in serial sections from OV, V, and NBE preincubated with RSA59-infected T mice. The following primary antibodies were used in immunohistochemistry (IHC) staining of brain, liver and spinal cord tissue sections: (a) 1:250 dilution of anti-Iba1 (Wako, Richmond, VA, USA) antibody to examine inflammation (microglia/macrophage upregulation); and (b) 1:50 dilution of a monoclonal antibody directed against the nucleocapsid protein (N) of MHV-JHM (monoclonal antibody clone 1-16-1 provided by Julian Leibowitz, Texas A&M University) to track the dissemination of viral antigen in brain and liver tissue sections. Bound primary antibodies were detected by an avidin-biotin immunoperoxidase technique (Vector Laboratories, Burlingame, CA, USA) using 3,3-diaminobenzidine as the substrate. Control slides from OV, and V mice were stained in parallel. All slides were coded and read in a masked manner. Stained sections were mounted in Refrax mounting medium, and observed under an upright light microscope (Nikon Eclipse 50i) and analyzed with Nikon imaging software (NIS, Nikon Corp, Tokyo, Japan).

### Quantification of Histological and Immunohistological Samples

Quantification of anti-viral staining and microglia-mediated inflammation in different neuroanatomic regions in the brain and spinal cord were adapted from previous methodology (Donnelly et al., 2009; Singh et al., 2019) and performed by Fiji (ImageJ 1.52g) software. Briefly, a threshold value was defined for each image to ensure that all labeled cells were selected. The magnitude of viral antigen staining and Iba1+ microglia/macrophage activation was defined as the percentage area of staining, i.e., [target stained area/total selected area)*100]. The entire quantification procedure was performed and read in a masked manner by more than one experimental investigators.

To quantify the areas with myelin loss (demyelination) in OV, V, and T mice of day 30 p.i., 3-4 LFB-stained spinal cord cross-sections from each mouse were randomly selected and analyzed using Fiji software (ImageJ 1.52g); the total number of mice in each group was 5 (*n* = 5). The total perimeter of the white matter regions in each cross-section was outlined and calculated by summing the dorsal, ventral and anterior white matter areas in each section. The total area of the demyelinated regions were also outlined and added for each section separately. The percentage of spinal cord demyelination per section per mouse was obtained by = (the total area of the demyelinating plaque/total area of the calculated white matter)*100 (McGavern et al., 1999; Singh et al., 2019).

### Data and Statistical Analysis

The study was not pre-registered. Mice were randomly assigned to each treatment group and experimentation. Data analyses were performed in a masked manner. Data normalization was undertaken to control for sources of variation of baseline parameters and to allow comparison of the magnitude of NBE effects in different conditions. For each experimental analysis, the level of significance and (mean ± SEM) values of independent experimental data points were plotted in scatter diagrams for “*n* = 5” (number of independent values) and mentioned separately in each figure legend. Technical replicates were only used to ensure the reliability of single values. Student’s unpaired *t-*test was used to identify significant differences in two-group comparisons. The tests were two-tailed. Multiple comparisons were achieved by RM one-way ANOVA test followed by Tukey’s multiple comparison test and Sidak’s multiple comparison test. For all experiments, statistical significance was set at a *P*-value of <0.05. All statistical analyses were carried out using GraphPad Prism 6.0 software (GraphPad Software, Inc). No sample calculation was performed.

## Results

### NBE Treated RSA59-Infected Neuro-2A Cells Shows Reduced Viral-Induced Cytopathy

**Figure 1:**
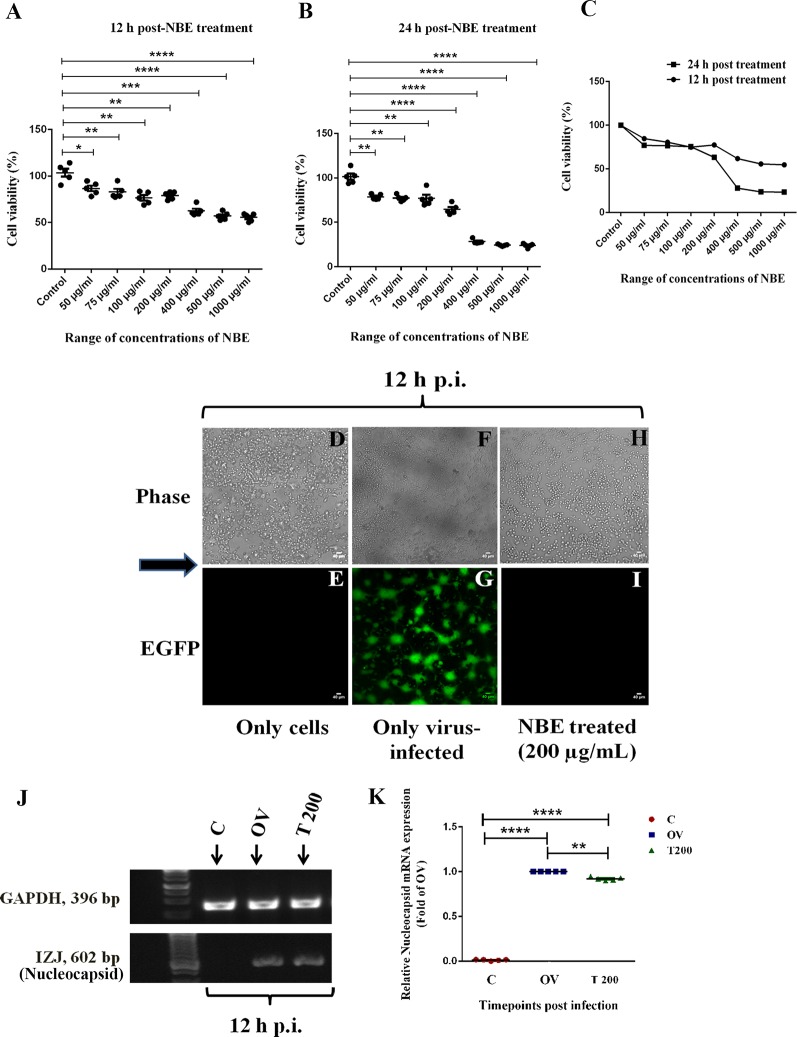
Neem bark extract (NBE) treated Neuro-2A culture restricts viral spread and cytopathy significantly. The cytotoxicity of NBE was checked by 3-(4,5-dimethylthiazol-2-yl)-2,5-diphenyl tetrazolium bromide (MTT) reduction assay in N2A cells. **(A–C)** MTT reduction assay in Neuro-2A cells estimated IC_50_ dose of NBE is 400 μg/ml both at 12 h (−38.25 ± 5.484, 12 h = ***p < 0.001; **A)** and 24 h p.i. (−72.03 ± 3.407, 24 h = ****p < 0.0001; **B)**. The Effective concentration (EC) range of NBE is 50–300 μg/ml. The mean values obtained from both time points were merged, are significantly different from respective control sets, and were line-plotted **(C)**. Epifluorescent microscopic analyses (10 × images) revealed significant inhibition of syncytia formation when Neuro-2A cells were post-incubated with NBE (200 μg/ml) upon RSA59 viral infection (at MOI of 1) at 12 h p.i. **(H,I)** as compared with OV cultures **(F,G)**, where characteristic RSA59-induced syncytia appeared. In parallel, Neuro-2A cells without RSA59 infection showed no syncytia formation in only-cells control **(D,E)**. Scale bar represents 40 μm. Effective reduction in the expression of viral nucleocapsid (IZJ) transcript was observed from post NBE treatment of RSA59-infected Neuro-2A cells (T 200) at 12 h p.i. in semi-quantitative PCR (0.07874 ± 0.02207, **p < 0.01, significantly different from OV using internal control GAPDH (P) **(J,K)**. Data represent mean ± SEM and level of significance was calculated by unpaired student’s *t*-test, followed by Mann–Whitney test and RM two-way ANOVA test, *p < 0.05; **p < 0.01; ***p < 0.001; ****p < 0.0001; and *n* = 5; C, Control (Only-cells); OV, Only-virus infected; T 200, NBE treated 200 μg/ml.

A Neuro-2A cell culture model was used to examine the potential of NBE to ameliorate viral-induced cytopathy and neuroinflammation. The cytotoxicity level of NBE to neuronal cells was evaluated. It showed significantly less cytotoxic effect on cell viability at a concentration that shows inhibitory activity upon RSA59 infection. MTT reduction assay revealed NBE concentration below 400 μg/ml was not significantly toxic to cells after 24 h of extract treatment. LC_50_ of NBE in Neuro-2A cells is 400 μg/ml and EC was chosen as 200 μg/ml for subsequent experiments (Figures 1A–C). Neuro-2A cells post-incubated with NBE following viral infection at MOI of 1 showed that NBE inhibited EGFP expressing syncytia formation and restricted viral spread entirely at 12 h p.i. (Figures 1D–I). Furthermore, significant changes in viral Nucleocapsid gene-level mRNA expression were observed at 12 h p.i. (Figures 1J,K; *p* < 0.05].

### NBE Treatment Blocks MHV-Spike Glycoprotein Mediated Cell-to-Cell Fusion

**Figure 2:**
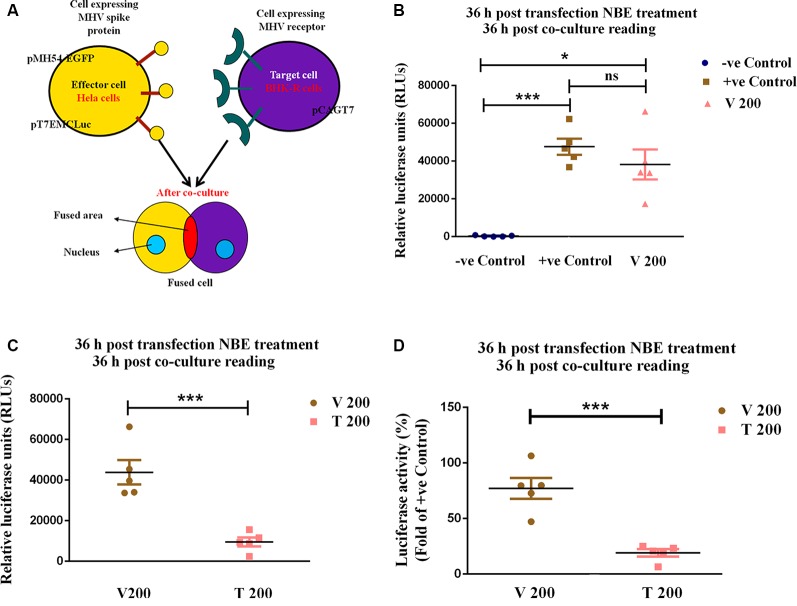
NBE significantly impairs mouse hepatitis virus (MHV)-Spike glycoprotein mediated cell-to-cell fusion. To assess the effect of NBE on spike glycoprotein-mediated cell-to-cell fusion, a virus-free luciferase reporter assay was performed. HeLa cells were considered as effector cells and BHK-R cells were target cells **(A)**. Relative Luciferase units (RLUs), as well as percentage luciferase activity, were determined using a luminometer and plotted. No significant difference in fusion efficiency was found in Vehicle control (V 200) compared to +ve control **(B)**. The fusion efficiency measured by the luciferase activity of 200 μg/ml NBE-treated, pMH54_EGFP_-expressing HeLa cells (effector cells) with pCAGT7-expressing BHK-R (target cells) was significantly reduced at 36 h **(C,D)** post-co-culture compared with untreated pMH54_EGFP_- and luciferase-expressing HeLa cells. Relative fold change was shown concerning OV **(C)**. Data represent mean ± SEM and statistical analyses were calculated by unpaired student’s *t*-test **(B,C**; ns = *p* > 0.05, *p < 0.05, ***p < 0.001, significantly different from V), and RM One-way ANOVA test followed by Tukey’s multiple comparison test **(D)**, where percentage luciferase activity of NBE treated sets were compared with positive controls; ***p < 0.001; significantly different from positive control sets; *n* = 5; −ve Control = (HeLa-pT7EMCLuc) + (BHK-R-pCAGT7), +ve Control = (HeLa-pT7EMCLuc + pMH54_EGFP_) + (BHK-R-pCAGT7), V 200 = (HeLa-pT7EMCLuc + pMH54_EGFP_) + (BHK-R-pCAGT7) + DMSO, T 200 = (HeLa-pT7EMCLuc + pMH54_EGFP_) + (BHK-R-pCAGT7) + NBE (200 μg/ml).

A virus-free cell-cell fusion assay based on luciferase activity was performed to examine the effect of NBE on spike glycoprotein-mediated intercellular fusion (Figure 2A). The co-transfected effector HeLa cells (pMH54_EGFP_ + pT7EMLuc) co-cultured with BHK-R target cells (pCAGT7) showed a high fusion capability at 36 h post- co-culture, and served as a positive control (non-NBE/DMSO treated). Vehicle (DMSO)-control showed no significant changes in luciferase activity compared to the positive control (Figure 2B). pMH54_EGFP_ and pT7EMLuc-expressing HeLa cells (effector cells) were treated with 200 μg/ml NBE treatment at 36 h post-transfection and co-cultured with pCAGT7-expressing BHK-R (target cells) cells. The fusion efficiency was measured after 36 h post-co-culture by luciferase activity. NBE significantly reduced cell-to-cell fusion in the treated set compared with vehicle, and positive control (Figures 2C,D; *p* < 0.05). Thus, the presence of NBE significantly inhibits spike protein-mediated viral entry and cell-to-cell fusion.

### RSA59 Preincubation With NBE Reduces Viral Replication *in vitro*

RSA59 virus stock was diluted in culture media (*in vitro*) followed by preincubation with DMSO (0.002% in the cell; <0.1%), which was used as vehicle control. Low-to-moderate concentrations of DMSO stimulate the yield of enveloped viruses specifically (Scholtissek and Müller, 1988; Rodríguez-Burford et al., 2003; Colucci et al., 2008). DMSO accelerates RSA59 infectivity and promotes viral assembly and viral replication *in vitro* compared with OV (Supplementary Figure S1). Throughout the study, the treated sets (T 200) were compared with infected-vehicle controls (V 200) to calculate significant changes in expression.

Viral induced cell-to-cell fusion and syncytia formation start from 7 h p.i. in Neuro-2A cells and NBE exhibited its most significant protective effect at 10–15 h p.i. Hence, all experimental analyses were shown at these time points throughout the study.

**Figure 3:**
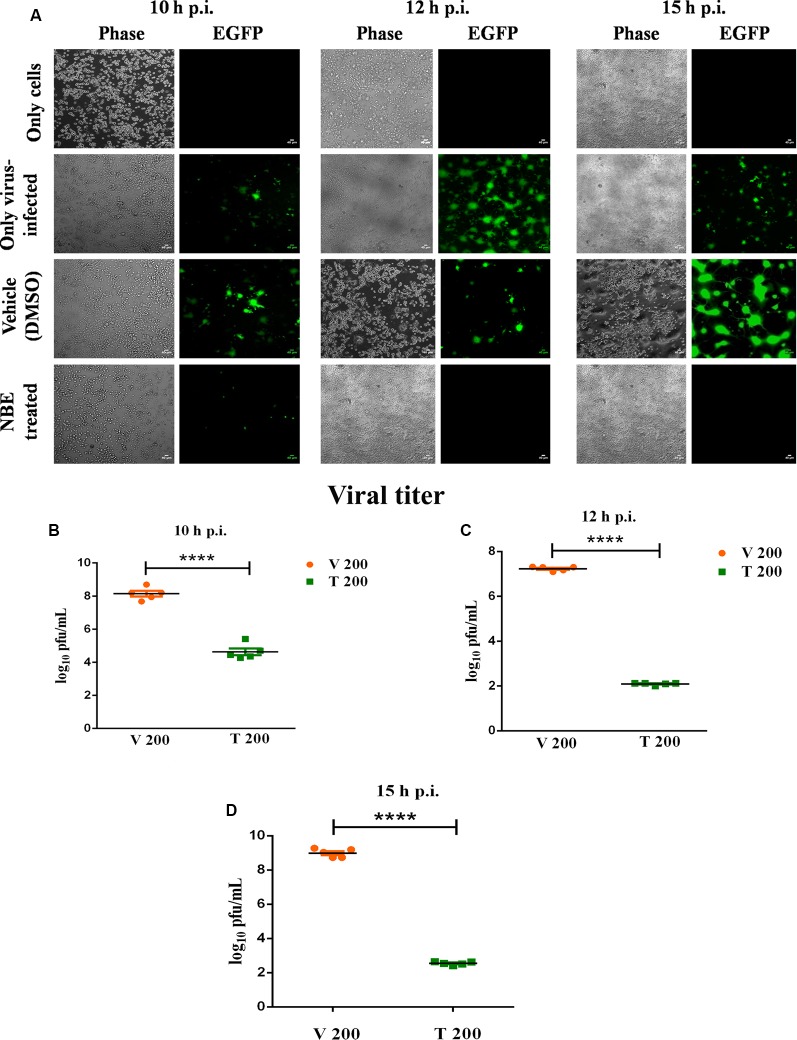
Preincubation of RSA59 with NBE inhibits viral replication significantly. Epifluorescent microscopic analyses (10 × images) revealed significant inhibition by NBE in syncytia formation at 10–15 h p.i. **(A)** compared to OV and V, where prominent viral-induced syncytia evolved. Scale bar = 40 μm. Neuro-2A culture supernatants following infection with NBE preincubated RSA59 virus (OV, V and T) at 10, 12, **(C)** and 15 h p.i. were subjected to comparative viral plaque assay on confluent monolayers of L2 cells (****p < 0.0001). Titers were expressed as log_10_ PFU/ml. NBE revealed significant regulation in RSA59 replication at 10–15 h p.i. **(B–D**; ****p < 0.0001). Data represent mean ± SEM in line-scatter plot and statistical significance was assessed by unpaired student’s *t*-test (****p < 0.0001, significantly different from V), *n* = 5, V 200 = Infected-vehicle (DMSO) 200, T 200 = NBE treated 200 μg/ml.

A significant inhibitory effect of NBE was shown in viral-induced cytopathy and the emergence of syncytia at 10–15 h p.i. (Figure 3A), when RSA59 was preincubated with NBE (200 μg/ml; T) or DMSO (V) before infecting Neuro-2A cells, and titer values were determined at 10, 12, and 15 h p.i. Media supernatants collected from OV (10^2^–10^7^diltution), T (Undiluted-10^5^ dilution) and V (10^2^–10^7^diltution)- infected Neuro-2A cells were tested for visible viral plaque formation in L2 monolayer cells. Viral titers of T were compared to V. NBE (T 200) showed a significantly lower (~10^4^–10^7^ times) titer, and restricted viral replication rate compared to V 200 at 10, 12, and 15 h p.i. (Figures 3B–D; *p* < 0.05). The differential replication kinetics could be attributed to the differences in virus-cell fusion between V and T.

### Preincubation of RSA59 Virus With NBE Downregulates the Expression of Viral Proteins *in vitro*

**Figure 4:**
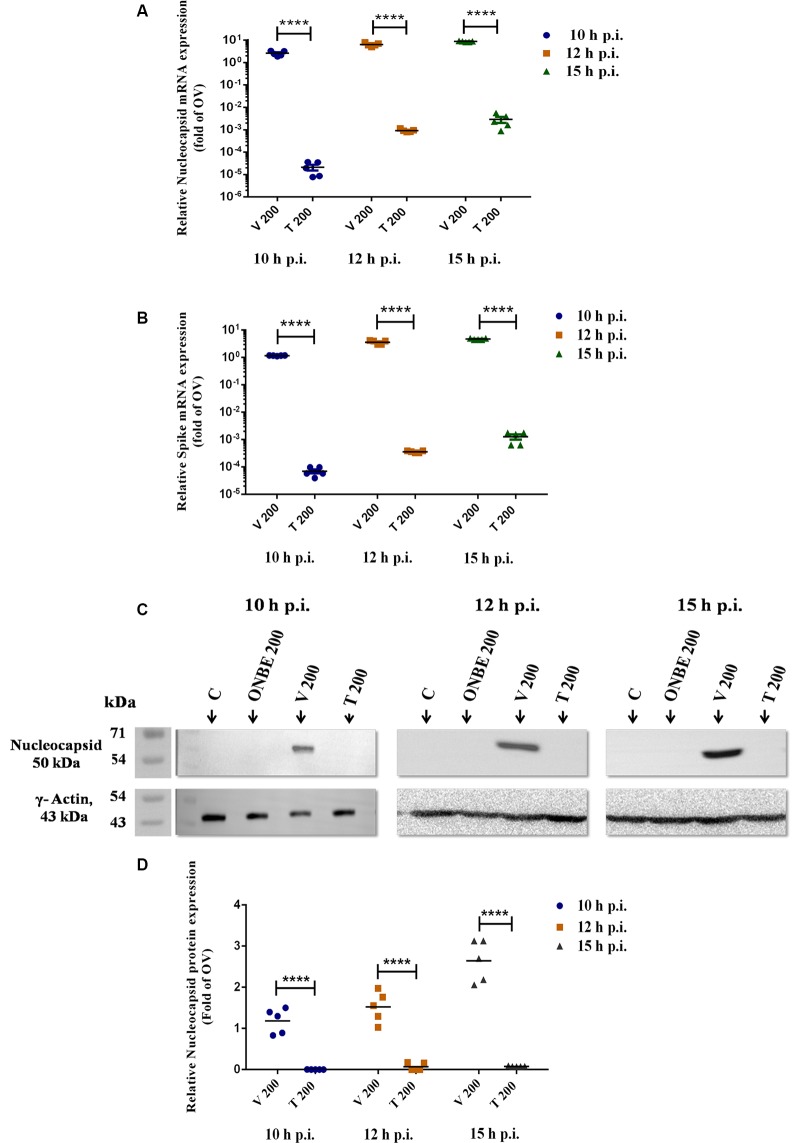
NBE preincubation with RSA59 reveals direct anti-viral activity *in vitro*. Real-time PCR analysis revealed that NBE downregulated expression of viral N protein at 10–15 h p.i. **(A**; ****p < 0.0001). Subsequently, the anti-viral regulation of NBE was also determined by the expression of viral S (mediator of virus-cell fusion) transcript at 10–15 h p.i. **(B**; ****p < 0.0001). Immunoblot analysis demonstrated a significant reduction in viral N protein expression upon NBE treatment compared to V at 10, 12, and 15 h p.i. **(C,D**; ****p < 0.0001). Relative fold changes were estimated with respect to OV. Results were normalized to GAPDH in RT-PCR and γ-Actin in immunoblot analysis as internal controls and expressed as mean ± SEM in scatter plots. Level of significance was calculated using RM two-way ANOVA followed by Tukey’s multiple comparison test (****p < 0.0001, significantly different from V); *n* = 5; C, Only cells (control); OV, Only-virus infected; ONBE, Only NBE (200 μg/ml); V 200, Infected-vehicle (DMSO) 200; T 200, NBE treated 200 μg/ml.

The anti-viral activity of NBE was examined in C, OV, V, and T sets at 10–15 h p.i. by analyzing the relative transcript levels of N and S genes using quantitative RT-PCR analysis. A basal level of mRNA and protein expression corresponding to the OV brain was considered as 1.0. The relative fold change of mRNAs in T was calculated and plotted in comparison to V. 200 μg/ml of NBE caused significant downregulation in the expression of viral transcripts, i.e., viral N (Figure 4A; *p* < 0.05), and S genes at the mRNA level (Figure 4B; *p* < 0.05). Also, a significant downregulation in viral N protein expression was observed at 10–15 h p.i. (Figures 4C,D; *p* < 0.05).

Our *in vitro* studies illustrated that NBE preincubation with RSA59 virions inhibits virus-induced virus-host attachment, cell-to-cell fusion, viral spread, viral replication, and downregulates viral N and S gene transcripts, and viral N protein synthesis (Supplementary Figure S3).

To take this study one step further, the possible direct anti-viral activity of NBE was investigated in a C57BL/6 murine model *in vivo*. From previous studies, it was depicted that day 6 p.i. is the peak of acute inflammation and viral titer and viral encephalitis are symbolized by microglial nodules and perivascular cuffing with a huge accumulation of inflammatory cells *in situ*. Hence, all *in vivo* experiments were done on day 6 and 30 p.i. to study the acute and chronic stage effect of NBE in neuropathogenicity, respectively.

### RSA59 Infected NBE-Treated Mice Reduces Body Weight

**Figure 5:**
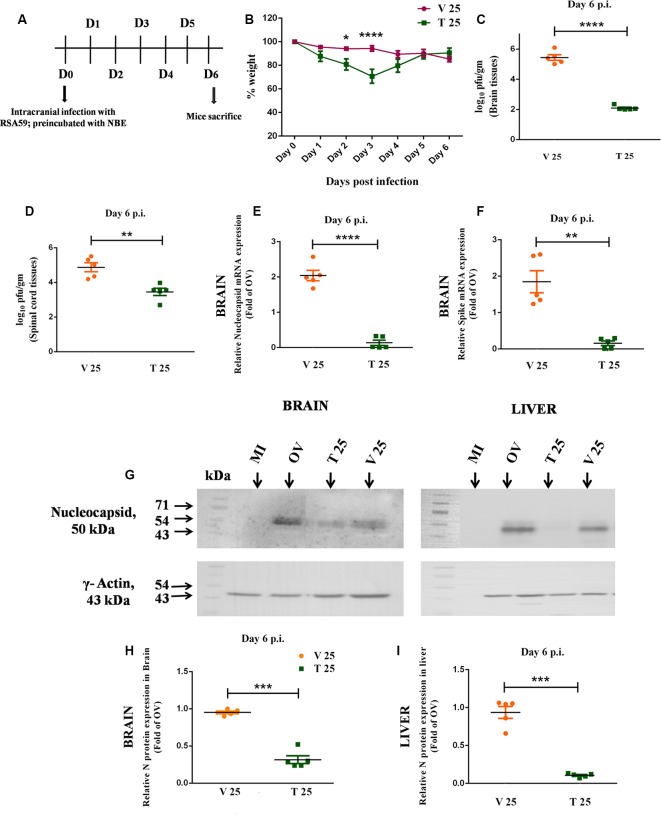
Intracranial infection in mice with RSA59, preincubated with NBE, restricted viral replication at day 6 p.i., implying a direct anti-viral property *in vivo*. Day-timeline of intracranial infection (i.c.) of C57BL/6 mice and sacrifice **(A)**. Mice were infected with RSA59 (20,000 PFU; OV), NBE-preincubated (25 mg/kg B.W.) RSA59 (T 25), and RSA59 preincubated with DMSO (V 25). The comparative effect of NBE on body weight change was shown upon i.c. inoculation with NBE-preincubated RSA59 into C57BL/6 mice brain. Significant changes were observed at day 2 and 3 p.i. in T 25 mice compared with V 25 mice **(B)**. Tissue lysates from the brain and spinal cord from OV, T and V mice were subjected to comparative viral plaque assay on confluent monolayers of L2 cells at day 6 p.i. Titers were expressed as log_10_ PFU/gm of tissue. T 25 revealed potential downregulation in RSA59 replication compared to V in the brain **(C)** and spinal cord **(D)**. The relative expression of viral N and S transcripts upon NBE treatment was determined in the brain tissues at day 6 p.i using qRT-PCR analysis. Results were normalized to GAPDH, and compared with OV. There is significant downregulation in N **(E)** and S **(F)** gene expression at the mRNA level during the acute infection stage. Similarly, mouse whole brain and liver lysates at day 6 p.i. were subjected to immunoblot analysis with Anti-N (Nucleocapsid) antibody at 1:50 dilution **(G)**. Significant downregulation of viral N expression at the protein level was observed in both brain **(H)** and liver **(I)** tissues. Results were normalized to γ-Actin, and compared with OV. Data represent mean ± SEM in scatter plots and statistical significance was determined by unpaired student’s *t*-test and RM Two-way ANOVA followed by Sidak’s multiple comparison test; *p < 0.05, **p < 0.01, ***p < 0.001, ****p < 0.0001, significantly different from V, *n* = 5; T 25 = NBE Treated 25 mg/kg B.W., V 25 = Infected-vehicle (DMSO) 25.

Mice were infected with RSA59 (20,000 PFU; OV), NBE-preincubated (25 mg/kg B.W.) RSA59 (T 25), and RSA59 preincubated with DMSO (V 25). Their body weights were recorded daily till day 6 p.i. (Figure 5A) and reported as mean ± SEM. There was no significant difference in initial body weights between T 25 and V 25 mice (0.0 ± 4.073, ns *p* > 0.05). At 2 days p.i. (13.34 ± 4.073, *p < 0.05) and 3 days p.i. (23.55 ± 4.073, ****p < 0.05) significant reduction in weight gain was observed in T 25 mice, but over time they regained the normal weight compared to V 25 mice at day 6 p.i. No significant changes were observed in T 25 and V 25 mice body weight at day 6 p.i. (−5.245 ± 4.073, ns *p* > 0.05; Figure 5B).

### NBE Preincubation Affects Viral Replication *in vivo*

To assess the potential of NBE to block viral replication, viral plaque assay was examined *in vivo*. Obtained titer values were expressed as log_10_ PFU/gm of tissue. Differential viral replication was determined by comparing T 25 (25 mg/kg B.W.) mice with V 25 in the brain and spinal cord tissues. There was a significant reduction in viral titer in T 25 tissue sets compared to V 25 in both brain (~1,000 times) and spinal cord tissues (~100 times) at day 6 p.i. (Figures 5C,D; *p* < 0.05). The obtained titer differences were statistically significant. Quantitative analyses on the effect of DMSO upon preincubation with the RSA59 virus showed no significant changes in V 25 compared to the OV brain (−0.5517 ± 0.2616), ns *p* > 0.05 and spinal cord (−2.006 ± 0.8510), ns *p* > 0.05.

### NBE Preincubated With RSA59 Reveals a Direct Anti-viral Property of NBE *in vivo*

*In vivo*, expression of viral N and S transcripts were examined in OV, V 25 and T 25 at day 6 p.i. using quantitative RT-PCR analysis. The relative fold change of mRNA in brains of NBE treated, T 25 mice were calculated in comparison to infected-vehicle control mice, V 25. A basal level of mRNA and protein expression corresponding to OV was considered as 1.0. V 25 brain tissues revealed a significant increase in N gene expression compared to OV brain tissues at the mRNA level (−1.041 ± 0.1476), **p < 0.01 at day 6 p.i. No significant changes were observed in S gene expression in V 25 compared to OV at the mRNA level (−0.8457 ± 0.3024), ns *p* > 0.05. I.C. inoculation of mice with RSA59 preincubated with NBE in the brain exhibited significant downregulation in viral N and S gene expression during acute infection (day 6 p.i.; Figures 5E,F; *p* < 0.05). Also, immunoblotting of the same brain samples revealed a significant downregulation in the viral N protein level at day 6 p.i. (Figures 5G,H; *p* < 0.05). Moreover, viral N protein expression upon NBE pre-treatment with RSA59 at 25 mg/kg B.W. dose declines in the liver at the acute stage of infection as well (Figures 5G,I; *p* < 0.05). No significant changes were observed in N protein expression in V 25 brain (0.04850 ± 0.01767), ns *p* > 0.05 and liver tissues compared to OV (0.06329 ± 0.07876), ns *p* > 0.05.

### RSA59 Preincubation With NBE Exhibits Altered Neuropathological Outcome in CNS Acute Infection

**Figure 6:**
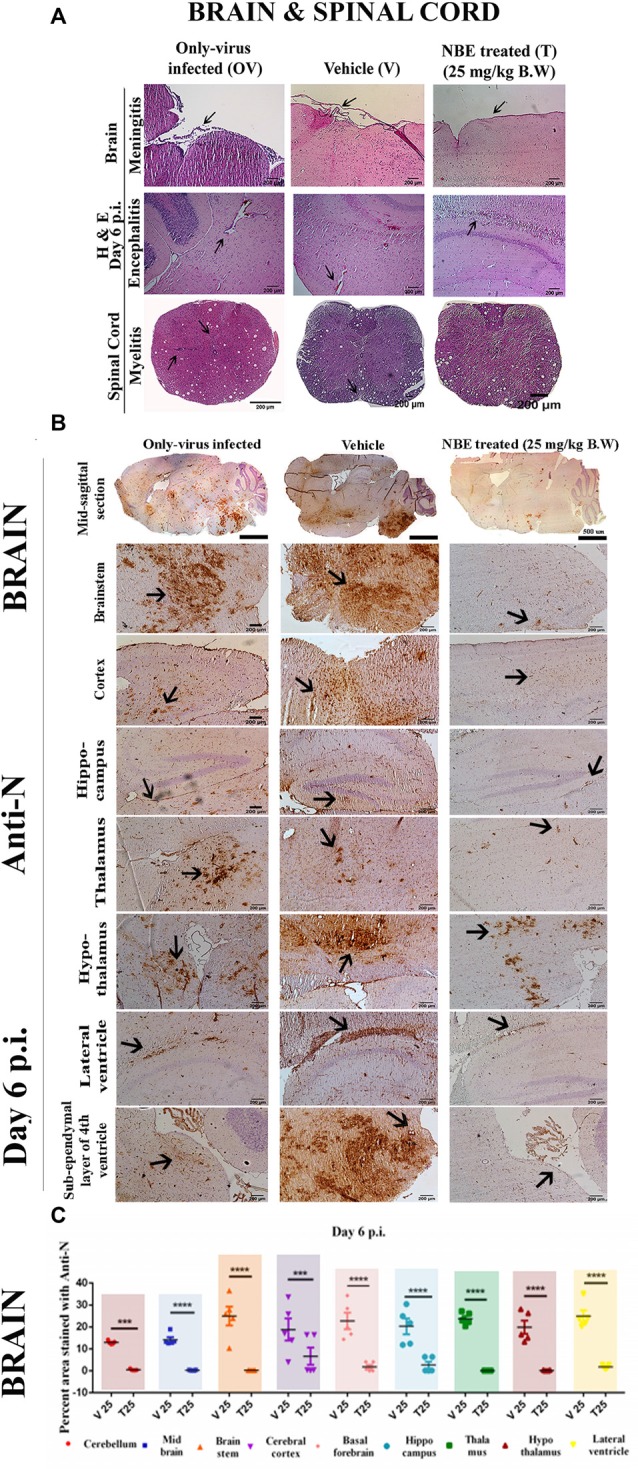
NBE prevents C57BL/6 mice from developing RSA59-induced encephalitis, meningitis, and myelitis at day 6 p.i. 5 μm mid-sagittal brain and spinal cord sections from OV, V and T mice at day 6 p.i. were stained with H and E and characteristic encephalitis and meningitis was observed in different regions of brain parenchyma in OV and V. Arrows indicate the inflamed regions (perivascular cuffing) and lymphocyte accumulation near to neurons. In comparison, NBE treatment revealed significantly less inflamed regions predominantly restricted in the lateral ventricular lining. Only-virus infected and infected-vehicle control spinal cord cross-sections at 6 p.i. stained with H and E exhibited the inflammatory cell distribution both in gray and white matter. No significant inflammation was observed in NBE treated spinal cord sections **(A)**. Serial mid-sagittal brain sections (5 μm thick) from OV, V and T sets of mice were immunohistochemically stained with anti-N antibody. At day 6 p.i. in OV, viral antigen distribution was observed almost throughout the brain including cortex, hippocampus, thalamus, hypothalamus, lateral ventricle, and brain stem. In contrast, in NBE treated brain sections, viral antigen was significantly restricted with less accumulation in different neuro-anatomic regions like the thalamus, hypothalamus, and the lateral ventricle/subventricular zone **(B)**. The mid-sagittal brain sections were divided into several neuroanatomical regions and the distribution of viral antigen in different neuroanatomical regions of the brain was quantified **(C)** by Fiji (ImageJ 1.52g) software. At acute stage (day 6 p.i.), NBE preincubation (25 mg/kg B.W.) significantly reduced viral antigen in cerebellum (***p < 0.001), midbrain (****p < 0.0001), brain stem (****p < 0.0001), ventral striatum/basal forebrain (****p < 0.0001), cerebral cortex (***p < 0.001), hippocampus (****p < 0.0001), thalamus (****p < 0.0001), hypothalamus (****p < 0.0001) and the lateral ventricle/subventricular zone (****p < 0.0001). Level of significance was determined by RM Two-way ANOVA followed by Sidak’s multiple comparison test; (***p < 0.001, ****p < 0.0001); significantly different from V sets, *n* = 5 mice/each experimental group; T 25 = NBE treated 25 mg/kg B.W.; V 25 = Infected-vehicle (DMSO) 25.

Histopathological studies with Hematoxylin and Eosin staining in NBE treated (T) brain and spinal cord tissue sections manifested a reduced severity of meningitis, encephalitis in the brain and reduced degree of myelitis in spinal cord tissues at day 6 p.i. (Figure 6A). In OV and V mice, encephalitis was characterized by perivascular cuffing and accumulation of inflammatory cells in brain parenchyma including lateral ventricle and mid-brain regions, whereas 25 mg/kg B.W. NBE treatment significantly reduced inflamed areas in brain parenchyma. Meningitis was characterized by the accumulation of inflammatory cells and viral antigen+ cells in OV and V, as described below. Intact myelin sheath with distinct gray and white matter regions were observed in NBE treated spinal cord compared with few inflamed areas with myelin loss in OV and V spinal cord tissues. Besides, to examine the tissue-specific effect of NBE, histopathological changes were also investigated in the MHV-tropic region and non-CNS tissue, i.e., liver. NBE treatment reduced the degree of non-necrotizing hepatitis with mild to single focal area in liver tissues compared to OV and V, characterized by huge number of necrotizing and non-necrotizing hepatic lesions. No characteristic anti-N or Iba1-stained lesions were observed in T liver tissues (Supplementary Figure S2).

### Diminished Viral Antigen Dissemination Was Observed in NBE Treated Mouse Brain Parenchyma During Acute Infection

Serial, mid-sagittal brain sections from OV, V, and T sets of mice at day 6 p.i. were investigated for viral antigen distribution in different neuroanatomic regions of the brain. Immuno-histostaining with anti-N revealed that viral antigen was well-disseminated throughout the brain, including cortex, hippocampus, thalamus, hypothalamus, lateral ventricle and brain stem in OV and V mice. On the contrary, viral antigen was significantly restricted with less accumulation in lateral ventricle/subventricular zone, cortex, hypothalamus and brainstem in NBE treated (25 mg/kg B.W.) brain sections. NBE contributed to restricted viral antigen spread in the brain (Figure 6B). The viral antigen distribution of OV, V, and T mice were quantified and compared among different neuroanatomic regions of the brain. At acute stage (day 6 p.i.), NBE preincubation (25 mg/kg B.W.) revealed a significant reduction in each neuroanatomical region of the brain, including cerebellum, midbrain, brain stem, ventral striatum/basal forebrain, cerebral cortex, hippocampus, thalamus, hypothalamus, and lateral ventricle. The mean difference ± SEM values of the difference in viral antigen spread were plotted as a scatter diagram (Figure 6C; *p* < 0.05). There are no significant changes observed in viral antigen distribution in V mouse brains (infected-vehicle) compared to OV mouse brains, except basal forebrain (−7.632 ± 3.039), *p < 0.05 and thalamus (−7.708 ± 3.039), *p < 0.05 regions.

### Protective Effect of NBE on RSA59-Induced Microglial-Mediated Inflammation in Mouse Brain

Serial, mid-sagittal brain sections of day 6 p.i. (5 μm thick) OV, V, and T mice were immunostained with anti-Iba1 (microglia/macrophage marker) to detect the activation of microglia in RSA59-affected regions.

**Figure 7:**
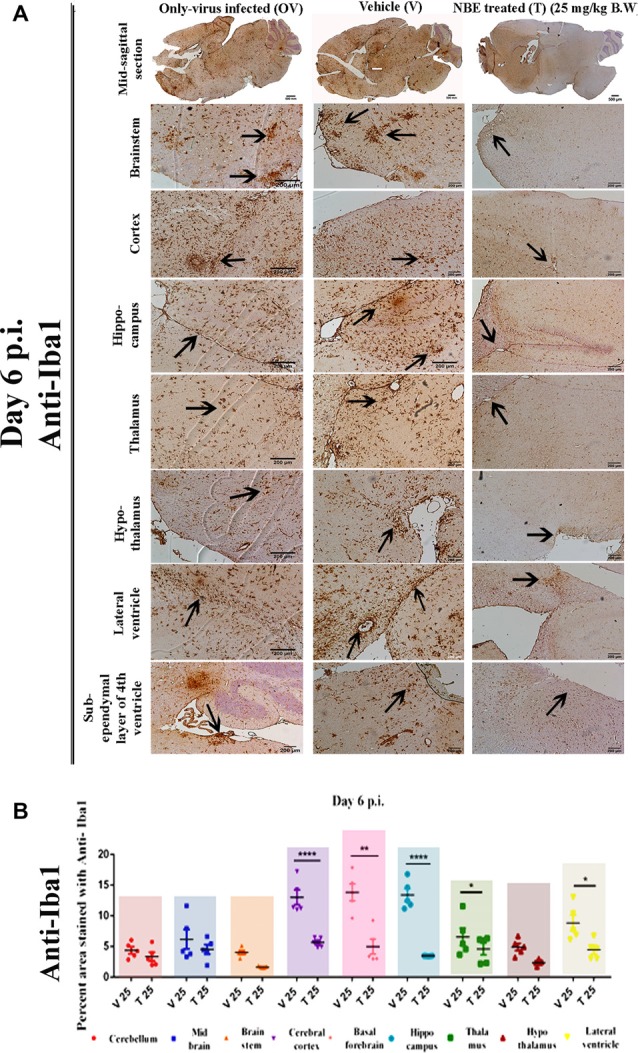
Potential effect of NBE on RSA59-induced microglial-mediated inflammation in the brain at the acute infection stage. Serial mid-sagittal brain sections from day 6 p.i. (5 μm thick) OV, V and T sets of mice were immunohistochemically stained with anti-Iba1 (microglia/macrophage marker). At day 6 p.i. NBE preincubated RSA59 infection showed a significant reduction in neuroinflammation in cortex, hippocampus, thalamus, hypothalamus, lateral ventricle, brain stem, and the sub-ependymal layer of the 4^th^ ventricle as compared with OV and V. Arrows indicate the inflamed region (microglia/macrophage with elongated nuclei) and microglial nodule formation in different neuroanatomical regions in the brain **(A)**. The distribution and level of inflammation was quantified. At day 6 p.i., significant differences were found upon 25 mg/kg B.W. NBE treatment in cerebral cortex (****p < 0.0001), basal forebrain (**p < 0.01), hippocampus (****p < 0.0001), thalamus (*p < 0.05), and lateral ventricle (*p < 0.05) as compared with V **(B)**. RM Two-way ANOVA followed by Sidak’s multiple comparison test was performed to determine the statistical significance (*p < 0.05, (**p < 0.01, ****p < 0.0001) significantly different from V, *n* = 5/each experimental group; T 25 = NBE treated 25 mg/kg B.W.; V 25 = Infected-vehicle (DMSO) 25.

In OV, characteristic activated microglia were present in the inflamed regions throughout the brain parenchyma. RSA59 preincubation with NBE showed the potential to fight MHV-induced neuroinflammation as there were very few resident non-activated microglia/macrophages present in the brain parenchyma. At day 6 p.i., NBE preincubated RSA59 infection showed a significant reduction in microglial activation in different neuroanatomic regions (Figure 7A), including cortex, hippocampus, thalamus, hypothalamus, lateral ventricle, brain stem, and the sub-ependymal layer of the 4th ventricle, as compared with OV and V. Arrows indicate the inflamed regions (microglia/macrophage with elongated nuclei) and microglial nodule formation in different regions. The distribution and level of inflammation were quantified. At day 6 p.i., a significant reduction in activated microglial distribution was found upon 25 mg/kg B.W. NBE treatment in cortex, basal forebrain, hippocampus, hypothalamus, lateral ventricle, and brainstem. The mean differences ± SEM values of the difference in different neuroanatomic regions were plotted as a scatter diagram (Figure 7B; *p* < 0.05). No significant changes were found in microglial distribution between V and OV mice (ns *p* > 0.05).

### Preincubation of RSA59 With NBE Restricts Viral Spread, and Microglia Activation in the Spinal Cord, and Protects C57BL/6 Mice From Myelin Loss

**Figure 8:**
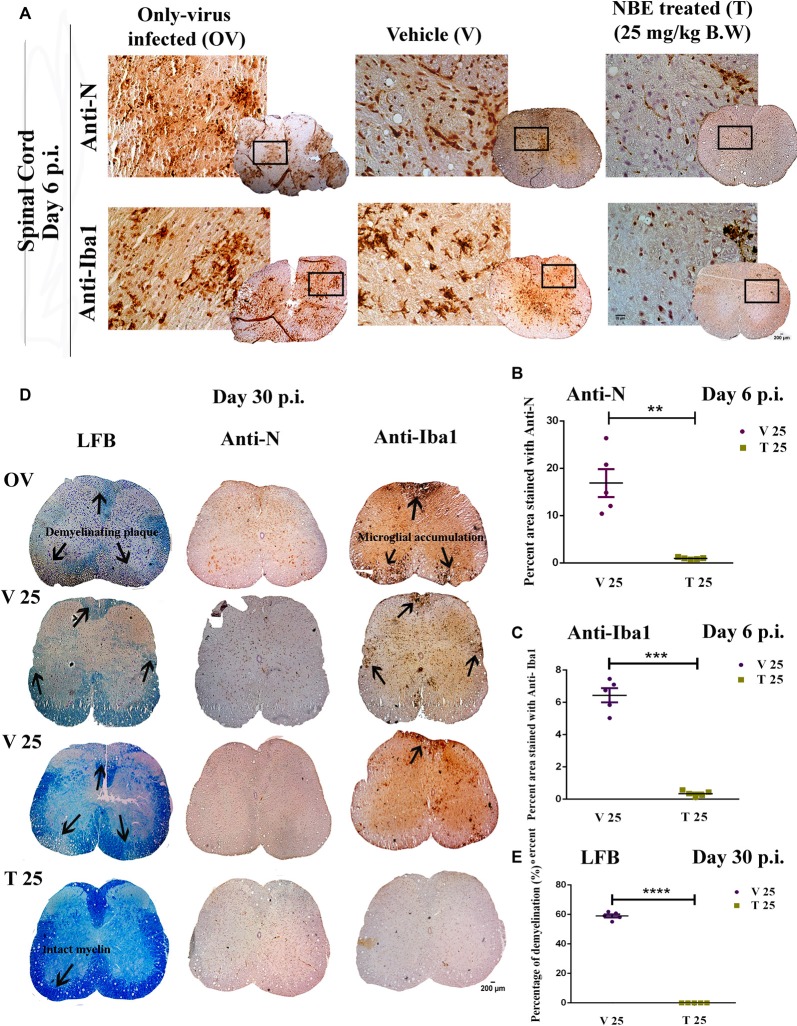
NBE restricts viral antigen distribution and microglial accumulation in the spinal cord and protects C57BL/6 mice from myelin loss. OV, V and T spinal cord cross-sections at day 6 p.i. were stained with anti-N antibody to study viral antigen distribution, anti-Iba1 antibody for microglia/macrophages, and day 30 p.i. cross-sections were stained with LFB to detect demyelination **(A)**. The total viral antigen-positive areas and Iba1-stained areas from day 6 p.i. sections were quantified and T mice were compared to V and plotted in a scatter diagram. Viral antigen distribution was significantly increased in the white matter as well as in the gray-white matter junction in OV and V; whereas, very few viral Nucleocapsid stained cells were observed in T **(B)**. Microglial activation and nodule formation were visualized throughout the OV and V spinal cord sections but there were significantly fewer Iba1+ cells distributed in T spinal cord white matter **(D)**. At day 30 p.i., no myelin loss was observed in NBE treated spinal cord sections from T sets of mice. In contrast, demyelinating patches characterized by extreme loss of myelin sheath were observed at serial levels of the spinal cord from OV and V sets of mice. The corresponding serial cross-sections were immunohistochemically labeled with anti-N and anti-Iba1 to observe the presence of viral-induced microglia/macrophage-mediated inflammation. OV and V sections showed the accumulation of Iba1+ cells in the corresponding demyelination patches whereas, in T mice, almost no Iba1+ cells were found **(C)**. The total viral antigen N+ regions **(B**; **p < 0.01), Iba1+ regions **(D**; ***p < 0.001), and demyelination **(E**; ****p < 0.0001) in spinal cord cross-sections were quantified and plotted in scatter diagrams. Level of significance was determined by unpaired student’s *t*-test, significantly different from V, *n* = 5/each experimental group, OV = Only-virus infected, T 25 = NBE treated 25 mg/kg B.W.; V 25 = Infected-vehicle (DMSO) 25.

OV, V and T spinal cord cross-sections from cervical and thoracic regions at day 6 p.i. were stained with anti-N antibody to study viral antigen distribution, anti-Iba1 antibody for microglial activation. Cross-sections were stained with LFB to detect demyelination at day 30 p.i. (Figures 8A,C). At day 6 p.i. the degree of viral antigen dissemination and microglial proliferation was significantly higher in white matter and gray-white matter junction in OV and V, but in T viral antigen was almost cleared and significantly less accumulation of Iba1+ cells was observed in white matter. Data from LFB-stained cross-sections from different levels of the spinal cord revealed that OV mice developed prominent demyelination. In contrast, the degree and amount of demyelination were significantly reduced compared to OV in T mice. OV and V mice showed a similar pattern and degree of demyelination (Figure 8C). The total viral antigen+ areas and Iba1-stained areas from day 6 p.i. cross-sections were quantified, and treated sets were compared with V and scatter-plotted (Figures 8B,C; *p* < 0.05). No significant changes were observed in viral antigen distribution (−0.2865 ± 3.992), ns *p* > 0.05 and microglial activation (−0.4799 ± 1.317), ns *p* > 0.05 in V spinal cord, compared to OV.

At day 30 p.i. (Figure 8D), no myelin loss was observed in NBE treated (T 25) spinal cord sections. In contrast, significant loss of myelin sheath resulting in demyelinating patches was observed in serial sections of different levels of the spinal cord from OV, and V 25 sets of mice. The corresponding serial sections, immunohistochemically labeled with anti-N and anti-Iba1, showed the accumulation of Iba1+ cells in the corresponding demyelination patches of OV and V-infected sections whereas, in T (T 25) mice, almost no Iba1+ cells were found to be morphologically activated. Quantification of demyelinating areas was performed and significant reduction was shown upon NBE preincubation with RSA59 in treated spinal cord sections (Figure 8E; *p* < 0.05), mean ± SEM values were plotted as a scatter diagram. No significant changes were observed in the quantification of demyelination in V mouse spinal cords compared to OV mice (−6.274 ± 2.645), ns *p* > 0.05.

### NBE Modulates Expression of Neuroinflammatory Markers *in vitro* and *in vivo*

**Figure 9:**
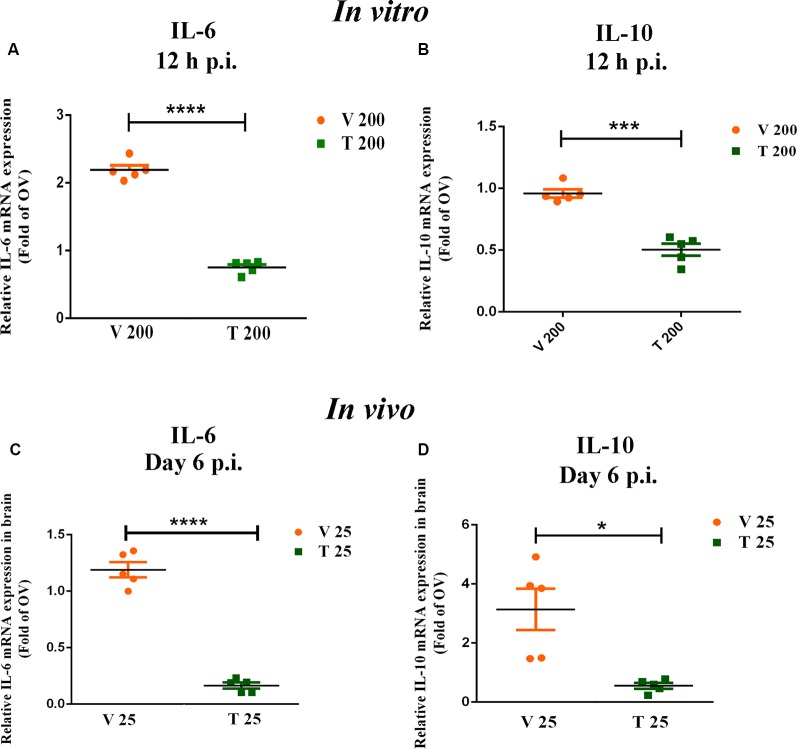
NBE pre-treatment modulates the expression of pro-inflammatory cytokines both *in vitro* and *in vivo*. NBE revealed potent anti-neuroinflammatory properties in MHV-induced acute inflammation. qRT-PCR data showed significant downregulation of proinflammatory cytokines IL-6 and IL-10 at 12 h p.i. when NBE (200 μg/ml) was preincubated with RSA59-infected Neuro-2A cells, as compared with infected-vehicle controls. A basal level of cytokine mRNA expression corresponding to only virus-infected (OV) sets was considered as 1.0. The mean ± SEM values were merged for both inflammatory cytokines IL-6 **(A)** and IL-10 **(B)** and plotted separately. *In vivo*, qRT-PCR data show significant downregulation of IL-6 expression at day 6 p.i. in NBE (25 mg/kg B.W.) treated mouse brains compared to V **(C)**, while IL-10 expression patterns showed less significant differential expression at the mRNA level at 12 h p.i. *in vivo* **(D)**. Data were represent mean ± SEM and statistical significance was determined by unpaired student’s *t*-test analysis **(C,D)** and Two-way ANOVA followed by Sidak’s multiple comparison test **(A,B)**; *p < 0.05, ***p < 0.001, ****p < 0.0001, significantly different from V sets, *n* = 5; V 200 = Infected-vehicle (DMSO) 200, T 200 = NBE treated 200 μg/ml, T 25 = NBE treated 25 mg/kg B.W.; V 25 = Infected-vehicle (DMSO) 25.

Neuro-2A cells, infected with NBE-preincubated RSA59 (at MOI of 1) were analyzed for the potential of NBE in controlling the expression of proinflammatory cytokine IL-6 and inflammatory cytokine IL-10. The basal level of cytokine expression corresponding to OV was considered as 1.0. V 200 cells revealed a significant increase in IL-6 expression (−1.192 ± 0.06705), ***p < 0.001, compared to OV cells. Quantitative RT-PCR results revealed that IL-6 expression was significantly lower (~4 times) in T 200 cells compared to V 200 cells at 12 h p.i. (Figure 9A; *p* < 0.05). IL-10 expression patterns showed a significant reduction at the mRNA level in T 200 cells compared to V 200 cells (Figure 9C; *p* < 0.05).

*In vivo*, expression of pro-inflammatory IL-6 and inflammatory IL-10 were examined in V 25, T 25 and OV brain tissues at day 6 p.i. The basal level of expression corresponding to OV was considered as 1.0. Quantitative analyses showed no significant changes in IL-6 (−0.1891 ± 0.06729, ns *p* > 0.05) and IL-10 expression (−2.138 ± 0.6999, ns *p* > 0.05) in V 25 brain compared to OV brain. Quantitative RT-PCR results revealed that IL-6 expression was significantly decreased upon NBE treatment compared to the V brain (Figure 9B; *p* < 0.05). IL-10 expression also showed a significant reduction in T 25 compared to V 25 brains at day 6 p.i. (Figure 9D; *p* < 0.05).

*In vivo* studies demonstrated the potent anti-viral, and anti-neuroinflammatory effect of NBE, where NBE decreases viral replication, viral transcription, viral N protein synthesis, expression of inflammatory cytokines IL-6 and IL-10, viral-induced acute stage hepatitis, encephalomyelitis, and chronic stage neuroinflammatory demyelination (Supplementary Figure S3).

### Intraperitoneal Administration of NBE Exhibited a Significant Reduction in Viral Nucleocapsid Protein Expression

**Figure 10:**
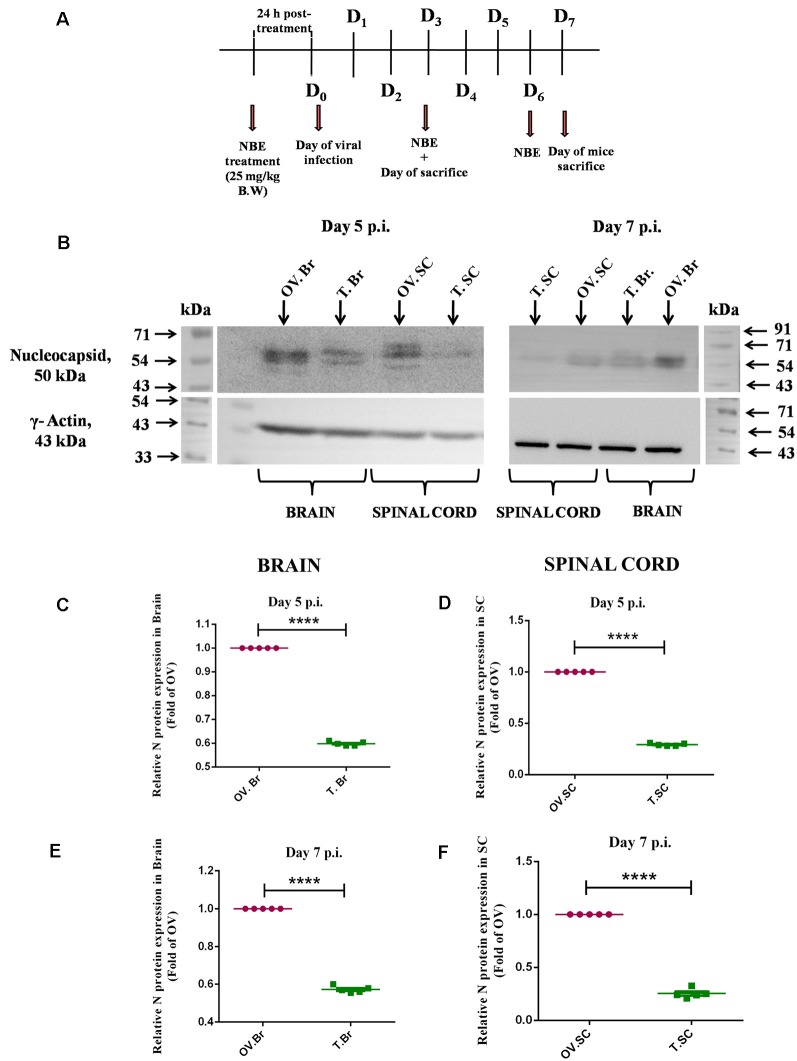
Intraperitoneal administration of NBE exhibited a significant reduction in viral Nucleocapsid protein expression. Day-timeline of intracranial infection (i.c.) following intraperitoneal (i.p.) administration of NBE into C57BL/6 mice and sacrifice **(A)**. Mice were infected with MHV-A59 (2,000 PFU; OV) and NBE (25 mg/kg B.W.) was administered i.p. following the MHV-A59 infection (T). Mouse whole brain and liver lysates at day 5 p.i. and day 7 p.i. were subjected to immunoblot analysis with Anti-N (Nucleocapsid) antibody at 1:50 dilution **(B)**. Results were normalized to γ-Actin, and compared with OV. At the acute stage of infection, NBE significantly decreased viral “N” protein expression in the brain **(B,C,E)** and spinal cord **(B,D,F)** proteins at both 5 and 7 days p.i. followed by i.p. NBE administration. Data were represented as mean ± SEM and statistical significance was determined by unpaired student’s *t*-test analysis; ****p < 0.0001, significantly different from OV sets, *n* = 5; OV.Br, Only-virus infected whole brain-isolated protein; T.Br, NBE (25 mg/kg B.W.)-treated whole brain-isolated protein; OV.SC, Only-virus infected whole spinal cord-isolated protein; T.SC, NBE (25 mg/kg B.W.)-treated whole spinal cord-isolated protein.

To check the therapeutic potential of NBE while administered intraperitoneally (i.p.), mice were given an i.p. injection of 25 mg/kg B.W. NBE in 100 μl 1 × PBS before 24 h of MHV-A59 (2,000 PFU, parental demyelinating virulent MHV strain) viral infection (Figure 10A). The drug was continued to administer once in every 3 days interval following i.c. injection. Mice were sacrificed at day 5 and 7 days p.i. to check the differential changes in the expression of viral nucleocapsid protein (N) in the brain, and spinal cord tissues.

It was evident from the immunoblot analysis that NBE showed tissue-specific potential effect upon i.p. administration in mice. NBE significantly reduced the expression of viral N protein in the brain (Figures 10B,C,E, *p* < 0.05) and spinal cord (Figures 10B,D,F, *p* < 0.05) at day 5 and day 7 p.i. at acute stages of MHV-A59-induced neuroinflammation.

## Discussion

This work aimed to examine the mechanistic anti-viral and anti-neuroinflammatory function of *A. indica* (Neem) in the MHV-induced demyelinating murine model of human MS. We report methanolic extract of neem bark (NBE) as a potent virus-host cell fusion inhibitor with strong evidence of reduced viral replication and neuroinflammation.

*In vitro*, the Neuro-2A cell-line was treated with NBE (200 μg/ml) following RSA59 infection, and it inhibited virus-induced cell-to-cell fusion, syncytia formation and suppressed the expression of viral N transcript at 12 h p.i. significantly. One of our recent studies identified Spike protein as a sole mediator of cell-to-cell fusion, irrespective of the murine CEACAM1 receptor (Singh et al., 2019). Spike glycoprotein itself plays a crucial role in viral entry, virus attachment, and syncytia formation *in vitro*. NBE is known for its enormous anti-inflammatory properties. Therefore, it was imperative to see whether NBE can directly bind to Spike at the infection-initiation step, and determine whether virus-to-cell fusion efficiency can be inhibited using NBE. Cell-to-cell fusion is an essential mechanistic step for virus pathogenicity and the virus exploits it to disseminate from white matter to gray matter of the spinal cord causing demyelination at the chronic disease phase.

Virus attachment to cells, virus-to-host cell fusion, and the resultant cytopathic effects are essential pre-requisites for any virus to establish rapid and productive infection and spread from the entry-site before the immune system gets activated. Spike protein is responsible for virus-host attachment, cell-to-cell fusion, and viral pathogenicity. First, we transfected wild-type HeLa cells with pMH54-EGFP Spike construct (causes cell-to-cell fusion) and treated the cells with NBE. Virus-free cell-to-cell fusion luciferase assay convincingly indicated that NBE significantly blocks viral binding and cell-to-cell fusion efficiency *in vitro*, suggesting that Spike might have an active interaction with NBE which is weakening the viral-to-cell fusion process.

In the succeeding experiment, we pre-incubated RSA59 with NBE for 15 min at 4°C to examine the direct interaction between NBE and virus and assessed its potential effect in inhibiting viral adsorption and cell-to-cell fusion both *in vitro* and *in vivo*. Parallel studies were performed using only DMSO as vehicle control at the same volume and concentrations. Earlier reports have revealed that moderate concentrations of DMSO stimulate the yield of enveloped virus proteins, and viral replication. DMSO can enhance the assembly of virus particles, viral components, and also the maturation of infectious particles (Wallis and Melnick, 1968; Scholtissek and Müller, 1988). Likewise, we observed that preincubation of RSA59 (enveloped coronavirus) with DMSO also amplified viral replication, and viral N and S gene expression at mRNA and protein levels *in vitro*, as compared with RSA59-infected controls. This indicates that DMSO might interfere with host membrane integrity and loosen its rigidity, facilitating the interaction of virus envelope proteins to host membrane and virus assembly on such weak membranes. Consecutively in all experimental analyses, NBE treated sets were compared with infected-vehicle controls, and still, significant reduction was observed in viral N and S transcripts, N protein synthesis, and viral replication in treated sets.

The blocking activity of NBE on host-virus interaction revealed reduced RSA59-induced virus-host attachment, cell-to-cell fusion, and syncytia formation in Neuro-2A cells when virus was pre-incubated with NBE. NBE reduces the expression of viral N and S genes, viral protein synthesis, and viral replication, which is indicative of NBE interaction with spike glycoprotein.

From a pre-clinical standpoint, our studies also revealed significant effects of NBE *in vivo*, when we preincubated RSA59 with NBE for 40 min at 4°C followed by its i.c. administration in mice. NBE caused impaired viral infectivity as a consequence of restricted virus-to-cell fusogenicity. Interestingly, we noticed that NBE significantly lowers the severity of liver hepatitis, region-specific brain inflammation in terms of viral dissemination, microglial proliferation, and activation, as well as chronic stage spinal cord demyelination. NBE restricted viral antigen spread with minimal accumulation in the lateral ventricle, cortex, hypothalamus, and brainstem at day 6 p.i. RSA59 infected brains showed widely distributed Iba-1+ cells throughout the brain parenchyma, forming characteristic microglial nodules and perivascular cuffing. In contrast, NBE treated brains had significantly fewer Iba1+ cells in most of the neuroanatomic regions, including cortex, hippocampus, thalamus, hypothalamus, lateral ventricle, brain stem, and the sub-ependymal layer of the 4th ventricle, following the infection route of viral antigen distribution.

Correspondingly, there was a significant reduction in chronic stage neuroinflammatory demyelination in NBE treated (25 mg/kg B.W.) mouse spinal cords in comparison to only virus-infected mice, which showed characteristic patches of myelin loss in white matter regions of the spinal cord. Together, our findings strongly suggest that NBE may directly bind with the virus and impair its interaction with the cell either during entry or during cell-to-cell fusion, or both.

Microglia are the principal immune cells in CNS and guard the integrity of CNS against any pathological assault. On the one hand, they can attain a signature pro-inflammatory state during CNS infection directed towards clearing the pathogen; on the other hand, they also become anti-inflammatory to restore homeostasis when inflammation gets out of hand. In our study, we selectively chose to assess the mRNA expression of a proinflammatory cytokine IL-6 and a regulatory cytokine IL-10 *in vitro* and *in vivo* upon NBE treatment. Results showed that preincubation of virus with NBE significantly downregulated the expression of IL-6 at 12 h p.i. in Neuro-2A cells as well as in NBE treated mouse brains at day 6 p.i. IL-10 is a potent two-faced immunoregulatory cytokine, it can induce both pro- and anti-inflammatory responses depending on the secretory immune cells and inflammatory state. Literature studies revealed that IL-10 suppresses activated macrophage/microglia functions, as well as intracellular pathogen killing and stimulation of pro-inflammatory cytokines like IL-12, IL-8, IFN-γ (Mühl, 2013; Verma et al., 2016). But high production of IL-10 can be associated with several chronic infections and result in pathogen accumulation with undesired proinflammatory effects (Lauw et al., 2000). NBE preincubation with RSA59 illustrated significant suppression of the expression of IL-10 compared to infected-vehicle controls both *in vitro* (200 μg/ml) and *in vivo* (25 mg/kg B.W. NBE). This suggests a slight pro-inflammatory nature of IL-10 in RSA59-induced neuroinflammation, and NBE preincubation modulates its regulatory expression. Moreover, *in vivo* intraperitoneal treatment of NBE (25 mg/kg B.W.) to MHV infected mice significantly reduced the expression of viral Nucleocapsid protein at the acute stage of infection, indicating the potential therapeutic effect of NBE in MHV-induced neuroinflammation.

## Conclusion

A top-down experimental approach was applied by using *Azadirachta indica* crude bark extract (NBE) to assess its potential neuroprotective effect and avoid bias. Most studies with particular compound(s) rather than crude extracts in Neuropharmacological and Neuropharmaceutical fields remain controversial for information processing biases and results vary from system-to-system. Our top-down approach revealed that NBE suppresses MHV-induced neuroinflammation and neuropathogenesis by inhibiting cell-to-cell fusion and viral replication.

In light of the present study, it may be concluded that NBE acts as a direct anti-viral agent inhibiting viral entry and spread when the virus is incubated in it before infection, in MHV-induced acute and chronic neuroinflammation. NBE, at its working doses, was never toxic to the cells and did not show any adverse effects in mice. NBE thus may not have significant side-effects and can be considered as a natural therapeutic agent or nutraceutical. As a result, our findings suggest a therapeutic approach towards preventing virus-induced cellular fusogenicity, acute (meningoencephalomyelitis), and chronic phases of neuropathogenicity (demyelination). However, the current study is focused to use NBE as anti-viral against MHV by targeting the Spike protein. From mechanistic standpoints, infection with NBE-preincubated virus causes reduced viral entry, viral replication along with the inhibition of successive generation of replicating viral particles. As a result, NBE reduced the neuroinflammation and consecutive demyelination in the experimental mice model. Moreover, we have also explained in Figure 1 that NBE treatment upon viral infection in murine neuroblastoma cells reduced cell-to-cell fusion and the expression of viral Nucleocapsid gene at mRNA level. Similarly intraperitoneal injection with NBE (25 mg/kg B.W.) into mice before 24 h of infection and in every 3 days interval till day 7 p.i. revealed a significant reduction in viral N protein expression. So combining these preincubation and post-incubation studies, our hypothesis is to show that NBE restricts cell-to-cell fusion, viral spread, viral replication, and consecutive neuropathogenesis by directly binding to Spike protein. NBE ameliorates microglial activation and prevents the production of pro-inflammatory mediators. Thus, NBE may apply to the amelioration of other neurodegenerative diseases like AD and PD where neuroinflammation is an underlying mechanism through microglial activation. Further research is warranted examining NBE-mediated amelioration of microglial activation acting as a therapeutic/prophylactic treatment for brain homeostasis in AD and PD neuropathological conditions.

Current findings will be salient in dissecting the metabolite profiling of NBE by combining Physico-chemical assays like Preparative HPLC and LC-MS analyses to identify individual promising compounds or synergistic effects of multiple compounds and to isolate them. Subsequently, the active anti-viral constituents may be delivered intraperitoneally, and if they successfully cross the BBB, they may bind to the virus particles *in situ*. Also, NBE, as a potent anti-viral agent, may be used to expand the study of anti-viral mechanisms involving other Spike expressing human coronaviruses like SARS, SARS-CoV-2, OC43 and their intercellular spread, fusion, replication, and consecutive pathogenesis. Our research combined with our prior studies helps expand our understanding of how specific amino acid mutations in the Fusion Peptide of Spike protein may result in alterations of virus infectivity and suggests that this insight can be used for therapeutic purposes. In the long run, our study aims to combine *in vivo* and *in vitro* studies with the whole extract and to design the future *in silico* experiments to confirm the potent anti-viral and anti-neuroinflammatory binding component/compound(s) of NBE with virus-host attachment Spike protein. The hypotheses and outcomes including additional future research will help us to sketch the underlying mechanism of anti-viral and anti-neuroinflammatory properties by NBE-spike protein interaction for novel therapeutic interventions.

## Data Availability Statement

All datasets generated for this study supporting the conclusion are presented within the main article or in supporting information as supplementary figures.

## Ethics Statement

The studies involving animals were reviewed and approved by institutional animal care and use committee at the Indian Institute of Science Education and Research Kolkata (IISER-K). The animal protocols were adhered to the guidelines of the Committee for the purpose of control and supervision of experiments on animals (CPCSEA), India.

## Author Contributions

LS and JD originally conceptualized and designed the experimental protocol. LS performed all the wet lab experiments, analyzed data, prepared the figures and wrote the manuscript. RP and AS assisted in *in vivo* mouse work. JD provided the day-to-day supervision in the project, analyzed data with LS in a masked manner, helped in critically reviewing and re-writing the manuscript. All the authors have gone through the final manuscript and approve the submission.

## Conflict of Interest

The authors declare that the research was conducted in the absence of any commercial or financial relationships that could be construed as a potential conflict of interest.

## Abbreviations

BBB: Blood-brain barrier
B.W.: Bodyweight
C: Non-infected control
CCl_4_: Carbon tetrachloride
CNS: Central nervous system
DMSO: Dimethyl sulfoxide
EAE: Experimental Autoimmune Encephalomyelitis
EC: Effective concentration
EGFP: Enhanced Green Fluorescence Protein
I.C.: Intracranial
IHC: Immunohistochemistry
IL-6: Interleukin 6
IL-10: Interleukin 10
I.P.: Intraperitoneal
LC_50_: Lethal concentration 50% of maximum response
MHV: Mouse hepatitis virus
MNCC: Maximum non-cytotoxic concentration
MTT: 3-(4,5-dimethylthiazol-2-yl)-2,5-diphenyl tetrazolium bromide
N: Viral Nucleocapsid gene (IZJ)
NBE: Neem bark extract
NLD: Non-lethal dose
OV: Only-virus infected control
PFU: Plaque forming unit
P.I.: Post-infection
ROS: Reactive oxygen species
S: Viral Spike gene
T: NBE preincubated (treated)
V: Infected-vehicle control.
