# Supplementary material for: Azadirachta indica A. Juss Ameliorates Mouse Hepatitis Virus-Induced Neuroinflammatory Demyelination by Modulating Cell-to-Cell Fusion in an Experimental Animal Model of Multiple Sclerosis
Source: Front Cell Neurosci. 2020 May 12;14:116. doi: 10.3389/fncel.2020.00116 (PMC7236902; doi:10.3389/fncel.2020.00116)

Supporting Information

Azadirachta indica A. Juss ameliorates Mouse Hepatitis Virus-induced neuroinflammatory demyelination by modulating cell-to-cell fusion in an experimental animal model of Multiple Sclerosis

Lucky Sarkar[1*], Ravi Kiran Putchala[1], Abass Alao Safiriyu[1], Jayasri Das Sarma[1ǂ]

[1]Department of Biological Sciences, Indian Institute of Science Education and Research Kolkata, Mohanpur, Nadia- 741246, West Bengal, India

[ǂ] Correspondence should be addressed to:
Prof Jayasri Das Sarma
Department of Biological Sciences,
Indian Institute of Science Education and Research Kolkata, Mohanpur, Nadia- 741246,
West Bengal, India
E-mail address: dassarmaj@iiserkol.ac.in
Phone: +91-9748642423/ +91-917003514069

Supplementary figure S1: DMSO elevates the yield of enveloped virus (RSA59) particles *in vitro*

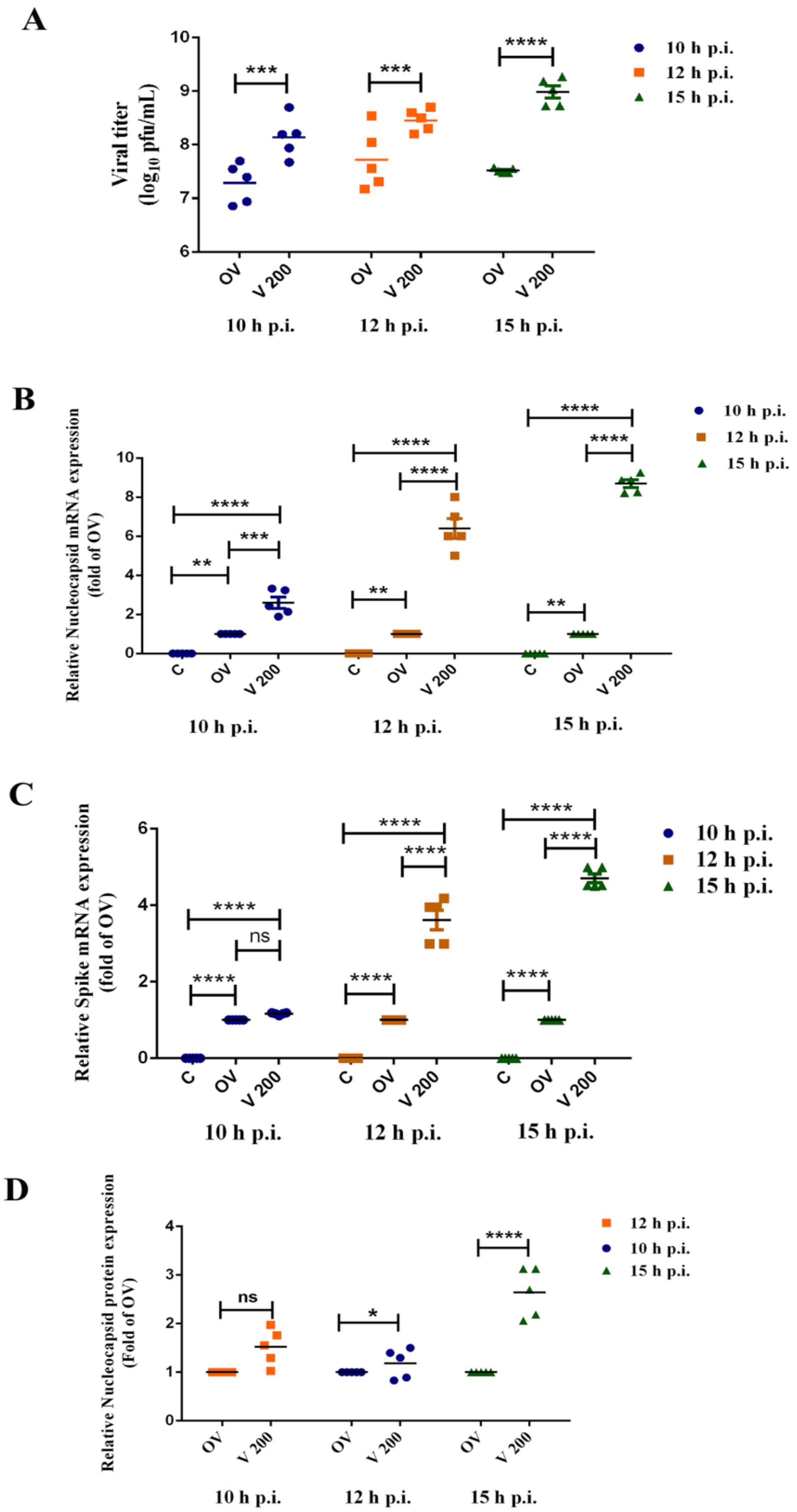

Figure S1| DMSO enhances RSA59 virus yield *in vitro*. RSA59 virus particles were preincubated with DMSO and Neuro-2A cells were infected. Culture supernatants collected from OV and V Neuro-2A cells at 12 h p.i. were subjected to viral plaque assay. DMSO promoted viral titer (A), increased viral N (B) and S (C) gene expression, and upregulated viral N protein expression (D) at 12 h p.i., compared to OV. Results were normalized to GAPDH in RT-PCR and γ-Actin in Immunoblot analysis, as internal control. Data represent mean ± SEM, relative fold changes were determined with respect to OV and statistical significance was established by RM one-way followed by Tukey's multiple comparison test (A, B & C), and Unpaired Student's t test (D); * $p<0.05$, ** $p<0.01$, ***, $p<0.001$, **** $p<0.0001$, ns $p>0.05$, significantly different from OV; n=5; C= Control (Only-cells), OV= Only-virus infected, V 200= Infected-vehicle (DMSO) 200.

Supplementary figure S2: Preincubation of RSA59 with NBE protects C57BL/6 mice from viral-induced hepatitis during acute infection

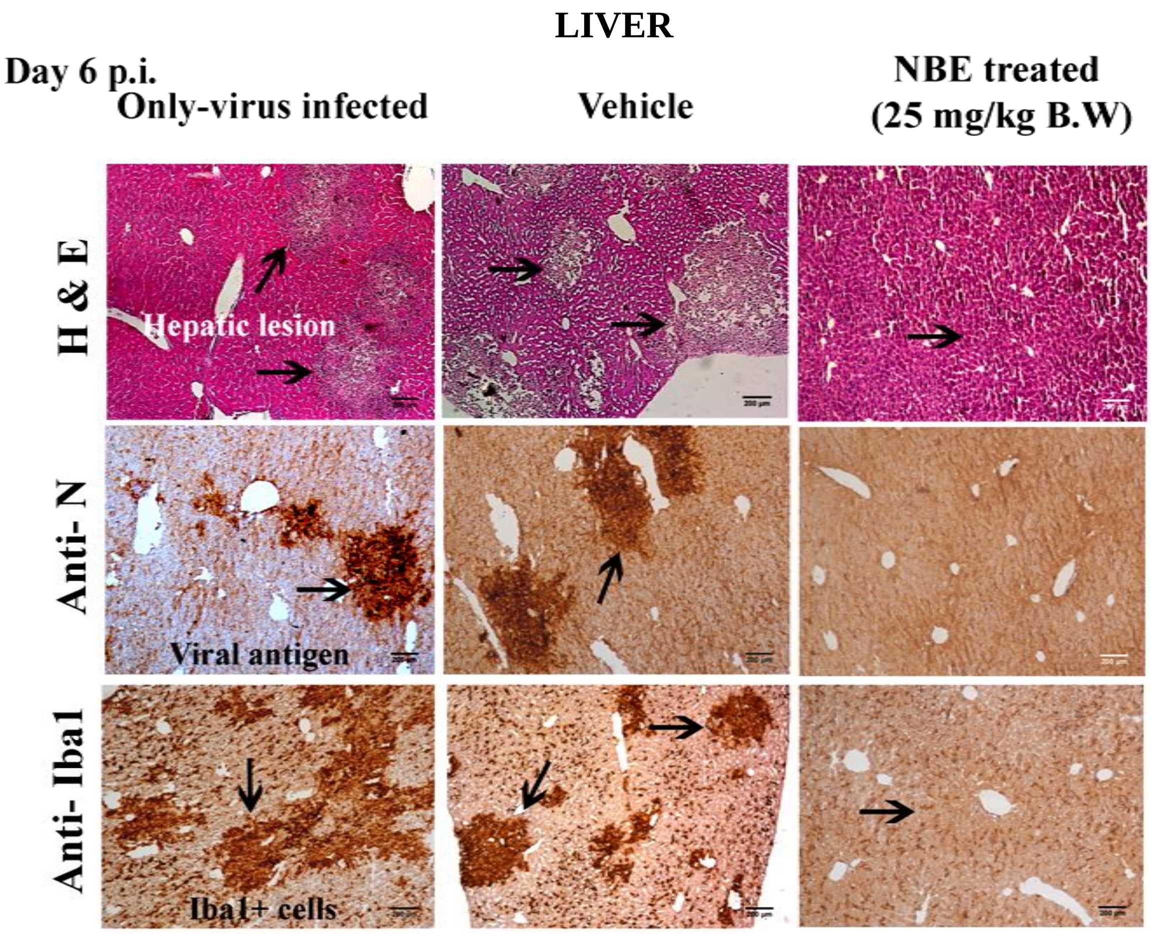

Figure S2: NBE prevents C57BL/6 mice from developing RSA59-induced hepatitis at day 6 p.i. In order to examine the tissue-specific effect of NBE, histopathological changes were investigated in MHV-tropic region and non-CNS tissue, i.e. liver. RSA59 was administered intracranially following preincubation with NBE at a dose of 25 mg/kg B.W. Liver tissues from day 6 p.i. stained with hematoxylin and eosin (H & E) showed moderate to severe hepatic lesions with presence of viral antigen (viral Nucleocapsid protein) and Iba1 (marker for activated microglia/macrophages)-positive cells within lesions in OV and V. 25 mg/kg B.W. NBE treatment showed increased anti-inflammatory properties including reduction in the degree of non-necrotizing hepatitis to only mild/single focal areas, less accumulation of viral antigen and significantly lower microglial/macrophage activation in liver sections compared to OV and V. Arrows indicate inflamed hepatic lesions in liver hepatocytes.

Supplementary figure S3: NBE impairs MHV-induced neuroinflammation

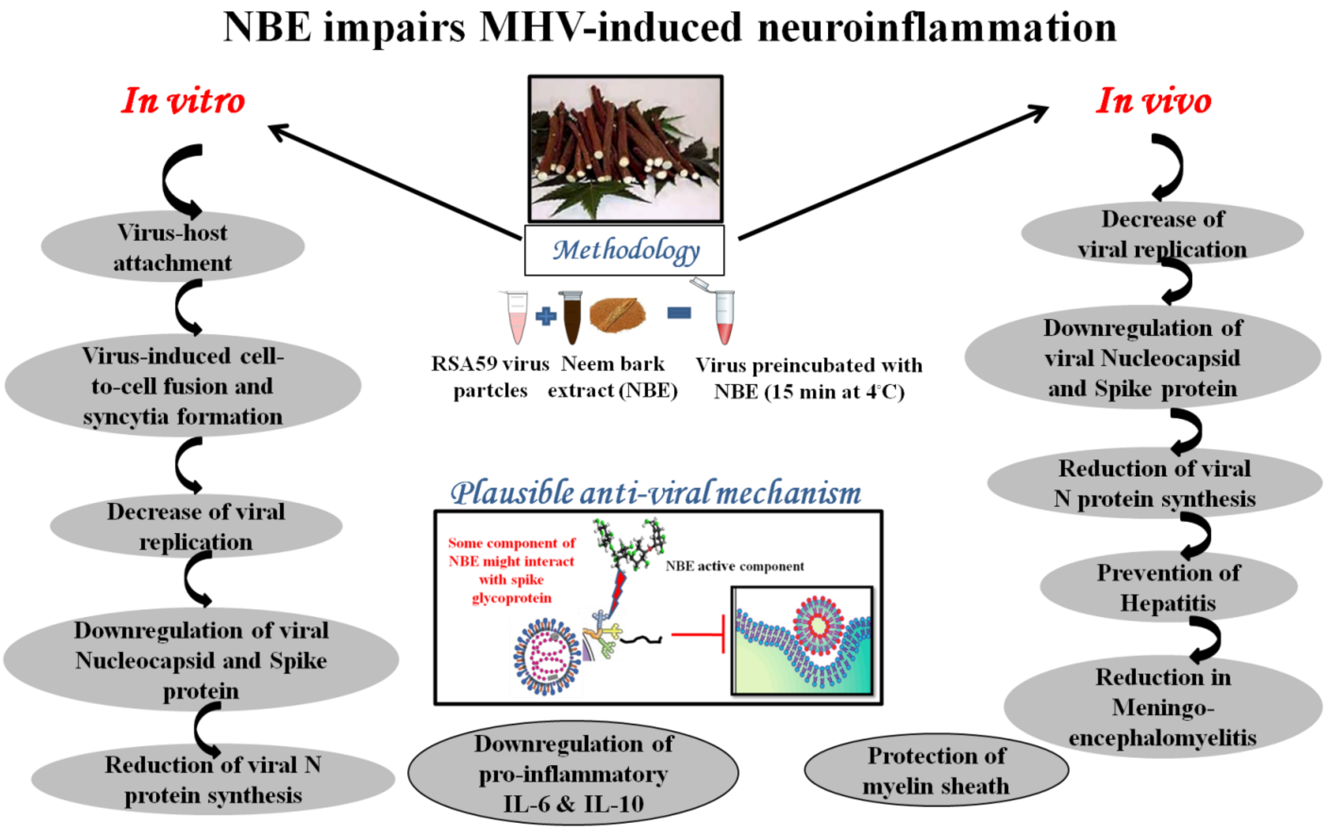

Supplement: Supplementary file 1 [file Presentation_1.pdf]
